# Mechanism Studies of Madden‐Julian Oscillation Coupling Into the Mesosphere/Lower Thermosphere Tides Using SABER, MERRA‐2, and SD‐WACCMX

**DOI:** 10.1029/2021JD034595

**Published:** 2021-07-02

**Authors:** Komal Kumari, Haonan Wu, Abigail Long, Xian Lu, Jens Oberheide

**Affiliations:** ^1^ Department of Physics and Astronomy Clemson University Clemson SC USA

**Keywords:** atmospheric tides, intraseasonal, Madden‐Julian oscillation, SABER, SDWACCMX, MERRA2

## Abstract

The Madden‐Julian Oscillation (MJO), an eastward‐moving disturbance near the equator (±30°) that typically recurs every ∼30–90 days in tropical winds and clouds, is the dominant mode of intraseasonal variability in tropical convection and circulation and has been extensively studied due to its importance for medium‐range weather forecasting. A previous statistical diagnostic of SABER/TIMED observations and the MJO index showed that the migrating diurnal (DW1) and the important nonmigrating diurnal (DE3) tide modulates on MJO‐timescale in the mesosphere/lower thermosphere (MLT) by about 20%–30%, depending on the MJO phase. In this study, we address the physics of the underlying coupling mechanisms using SABER, MERRA‐2 reanalysis, and SD‐WACCMX. Our emphasis was on the 2008–2010 time period when several strong MJO events occurred. SD‐WACCMX and SABER tides show characteristically similar MJO‐signal in the MLT region. The tides largely respond to the MJO in the tropospheric tidal forcing and less so to the MJO in tropospheric/stratospheric background winds. We further quantify the MJO response in the MLT region in the SD‐WACCMX zonal and meridional momentum forcing by separating the relative contributions of classical (Coriolis force and pressure gradient) and nonclassical forcing (advection and gravity wave drag [GWD]) which transport the MJO‐signal into the upper atmosphere. Interestingly, the tidal MJO‐response is larger in summer due to larger momentum forcing in the MLT region despite the MJO being most active in winter. We find that tidal advection and GWD forcing in MLT can work together or against each other depending on their phase relationship to the MJO‐phases.

## Key Points

- SABER and SD‐WACCMX diurnal temperature tides show a statistically similar response connected to the tropospheric Madden‐Julian Oscillation
- Tropospheric radiative and latent heating is more important in forcing the tidal MJO‐response than tropo/stratospheric wind‐filtering
- Coriolis, pressure gradient, advection, and gravity wave drag forcing are the important mechanisms in the MLT region for tidal MJO‐response

## 1 Introduction

Upward propagating atmospheric waves of tropospheric origin such as gravity waves (GWs), tides, Kelvin waves (KWs), and Rossby or planetary waves (PWs), significantly impact the dynamics and thermal state of the upper atmosphere above 60 km (Andrews et al., 1987; Fritts & Alexander, 2003; Liu, 2016; Oberheide et al., 2009), and its mean flow through energy and momentum deposition (Jones et al., 2014, 2016, 2019). Atmospheric tides, in particular, dominate the mesosphere/lower thermosphere (>60 km) large‐scale dynamics (Hagan et al., 2009; Oberheide et al., 2009) and are also important for ionospheric variability through E‐region dynamo action (e.g., Chang et al., 2013). A prominent example of tides forced in the troposphere is the DW1 (westward propagating migrating diurnal tide with zonal wavenumber 1) originating mainly from radiative forcing in the troposphere and the DE3 tide (eastward propagating nonmigrating diurnal tide with zonal wavenumber 3) that mainly originates from latent heat release in tropical deep convective clouds. Both tides propagate upward and introduce a large longitudinal and local time variability in the MLT region (Hagan et al., 1997; Hagan & Forbes, 2002), and impact the space weather of the ionosphere (Immel et al., 2006, 2009; Pedatella et al., 2016). Tides also propagate into the thermosphere, where they impact the energy budget of the thermosphere through modulation of the 15 µm CO_2_ and 5.3 µm NO infrared emissions (Nischal et al., 2017, 2019), mean winds, temperature, and constituents (Hagan et al., 2009; Jones et al., 2014, 2019; Oberheide et al., 2020).

The Fourier spectrum of tides shows variabilities over a wide range of temporal scales, from PW to intraseasonal to seasonal and longer interannual timescales (e.g., Kumari & Oberheide, 2020; Lu et al., 2011). The general scenario of the tropospheric tidal coupling to the MLT region on seasonal and longer time scales is well established (Oberheide et al., 2011; Wu et al., 2008). Recently, the focus has been on understanding the characteristics of the variability on intraseasonal (30–90 days) (e.g., Sassi et al., 2019) and smaller (<30 days) timescales (e.g., Dhadly et al., 2018; Kumari & Oberheide, 2020), which is now possible due to increased short‐term data availability throughout the atmosphere (Gan et al., 2017; Pedatella et al., 2016). The intraseasonal peak at 40–60 days in the Fourier spectrum of tidal diagnostics in the MLT region has been studied in relation to the tropical tropospheric Madden‐Julian Oscillation (MJO) (Gasperini et al., 2020; Kumari et al., 2020; Vergados et al., 2018; Yang et al., 2018). The MJO is the dominant form of intraseasonal variability in the tropical atmosphere (Madden & Julian, 1972), and it is characterized by large‐scale convective anomalies that develop over the tropical Indian Ocean and propagate slowly (∼5 m/s) eastward over the western‐central‐eastern Pacific with individual events lasting 30–90 days (e.g., Zhang, 2005). The MJO can be considered as an eastward propagating KW radiating away from the source region (Madden & Julian, 1994). Since the MJO is confined to the lower atmosphere (e.g., Tian et al., 2012; Zhang, 2005) due to its low frequency and slow zonal propagation speed, it may modulate the upward propagating tides and GWs and thus potentially induce the same periodic signatures across a broad range of vertical levels. Li and Lu (2020) found the modulation with respect to the locations of MJO‐convection (MJO‐locations or MJO‐phases) in GW temperature variances at SABER altitudes, while Yang et al. (2018) and Kumari et al. (2020) studied the modulation of intraseasonal signals in the MLT tides corresponding to the MJO‐locations from SD‐WACCM and SABER, respectively. Yang et al. (2018) performed the analysis only for boreal winter since MJO is mostly active during this season, while Kumari et al. (2020) found a strong response in all seasons depending on the MJO‐phase/location. The latter study found that the DE3 tidal response (∼25%) to MJO‐locations can be about two times as strong as that of DW1 in summer (∼8%) and winter (∼10%). Moreover, the seasonal variation of the intraseasonal variability at different MJO‐locations in nonmigrating tides is generally more prominent than in the migrating diurnal tide. Note that the tidal seasonal amplitude was removed in the study and the analyzed intraseasonal amplitude anomalies at different MJO‐locations were not overlaid on their own seasonal‐amplitude variations. Further, using model simulations, Gasparini et al. (2020) discussed the role of the DE3 tide and the quasi‐3‐day ultrafast KW (both excited by deep tropical tropospheric convection) in coupling tropospheric intraseasonal variability to the thermosphere.

Lieberman et al. (2007) proposed that whole‐atmosphere coupling involving tidal variability may occur via (i) direct amplitude modulation by tropospheric heating, (ii) zonal mean flow interactions that modulate the tidal behavior as waves propagate through a variable background in the middle and upper atmosphere, or (iii) nonlinear wave‐wave interactions. The MJO is known to modulate stratospheric GWs, GW drag on tides, and zonal winds (e.g., Alexander et al., 2018; Eckermann et al., 1997; Li & Lu, 2020; Lieberman, 1998). As such, any MJO modulations of the MLT tides can be imposed by an MJO in tropospheric forcing, tropo/strato/mesospheric winds, GW momentum forcing, and possibly other effects. Previous statistical analyses by Yang et al. (2018) and Kumari et al. (2020) do not enable the study of underlying physical mechanisms. Obtaining the latter is the main goal of the present study. We first extract the MJO‐like response in intraseasonal tidal variability and then we perform a comparative analysis of the MJO‐response in the MLT tides from SABER and SD‐WACCMX. We then conduct additional model simulations of tides with tropospheric MJO‐induced variability removed to separate forcing from wind filtering effects. This part of the study is focused on the years 2008–2010 which had several strong MJO events, the so‐called “year of tropical convection” (Waliser et al., 2012). We also quantify the relative contribution of individual terms in the tidal momentum equations to the MJO‐response in MLT tides: classical (Coriolis force and pressure gradient) versus nonclassical terms (advection and gravity wave drag [GWD]).

In Section 2, we discuss the use of multiple datasets to close the gap in our understanding of how MJO forcing in the troposphere manifests itself in tides in the MLT region. In Section 3.1, we show how to extract the MJO‐response in the intraseasonal variations at each of the MJO locations (phases 1–8) in MLT‐diurnal temperature tides using both observational and model database. Section 3.2 includes the comparative analysis of MJO‐response in model and observational tidal diagnostics. Section 3.3 includes the study of the MJO‐response in tropospheric tidal forcing, and Section 3.4 includes the wind filtering effect of zonal/meridional winds up to stratospheric altitudes. In Section 4, we diagnose and quantify each physical term responsible for contributing to the overall MJO‐response in the tidal momentum budget above stratospheric altitudes in the MLT region. We conclude in Section 5 with the findings and additional physical insights from this investigation.

## 2 Data

### 2.1 Temperature Diurnal Tides

The observational tidal baseline data used for the analysis is based on SABER short‐term tidal diagnostics followed by projection into symmetric and antisymmetric Hough Mode Extensions (HMEs). The SD‐WACCMX simulations of tides, including their projection on HMEs, are used for the comparative analysis with the observations.

#### 2.1.1 Sounding of the Atmosphere Using Broadband Emission Radiometry

**Figure 1:**
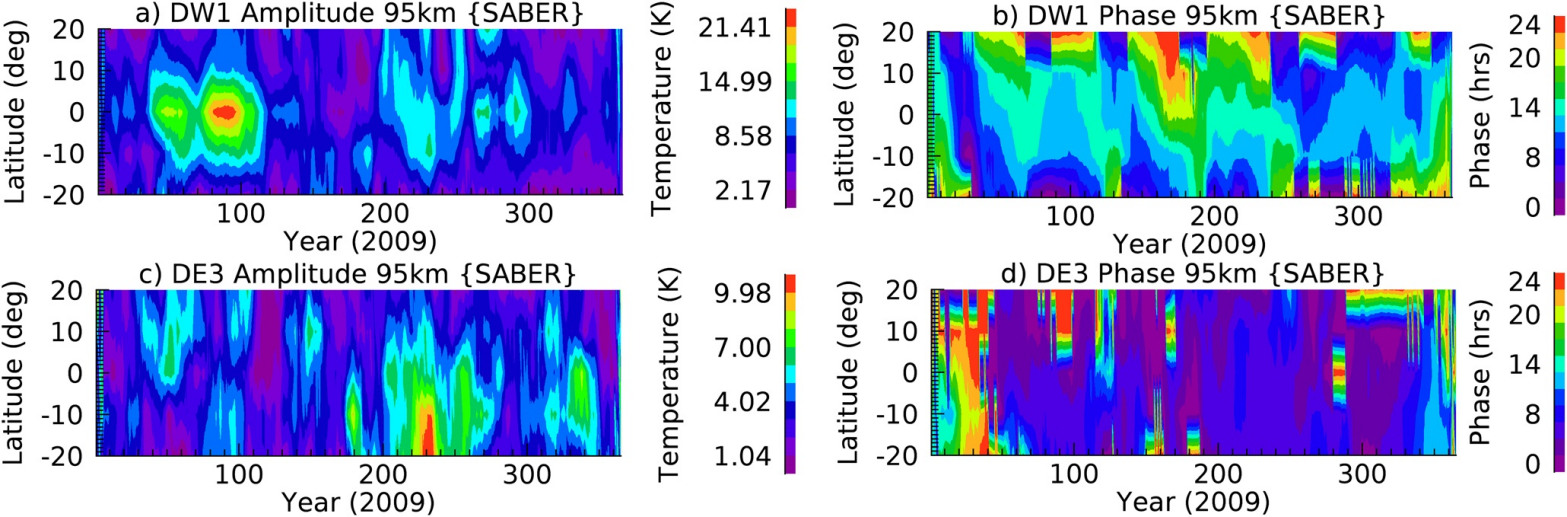
11‐day running mean of (a) DW1 and (b) DE3 tidal amplitudes and corresponding phases in (c) and (d) at 95 km observed by SABER during 2009. SABER, Sounding of the Atmosphere using Broadband Emission Radiometry.

Sounding of the Atmosphere using Broadband Emission Radiometry (SABER) (Russell et al., 1999) is one of the four instruments on the TIMED satellite. The instrument was designed to study the energy budget, chemistry, and dynamics of the middle and upper atmosphere, especially in the MLT region. SABER provides temperature profiles that are retrieved from the 15 and 4.3 μm CO_2_ channels (Rezac et al., 2015) and from which the temperature tides can be retrieved up to the MLT region (<110 km). We use version 2 of the current data to diagnose the time series of the diurnal migrating (e.g., DW1) and nonmigrating tides (e.g., DE3) from “tidal deconvolution” which is available from 2002 to 2019. Tidal deconvolution uses differences between observations taken during the ascending and descending portions of the satellite orbit. For a given zonal wavenumber, *k*, the daily difference between the ascending and descending nodes ($T_{k}$) is expressed as a linear combination of the eastward and westward propagating tides with zonal wave numbers *k −* 1 and *k* + 1, respectively. Exact solutions of the amplitudes and phases of the propagating tides can be obtained at the extrema of the $T_{k}^{2}$ vertical profile, and linear interpolation is used at the intermediate altitudes (see Oberheide et al. [2002] for complete mathematical details of the solution method). The amplitude error is ∼0.5 K in the MLT region due to the HME fitting. Figure 1 depicts the 11‐day running mean (for plotting purpose) of deconvoluted DW1 and DE3 tidal amplitudes along with the phases observed by SABER during 2009. Note that the winter (including early spring, December–March) to summer (including early fall, June–September) seasonal variations in DW1 and DE3 tides differ from each other. DW1 has maximum amplitude in spring while DE3 has in summer. In winter, DE3 has nonequatorial amplitudes which are smaller than amplitudes in summer months at equatorial latitudes. DW1 largely stays in equatorial latitudes with smaller amplitudes in summer than spring. This observational data set is identical to the one used in Kumari et al. (2020).

Previous studies have shown that SABER tidal deconvolution agrees well with data assimilation approaches (Lieberman et al., 2015; Pedatella et al., 2016) using NOGAPS‐ALPHA and WACCM + DART, respectively, and other general circulation model simulations such as eCMAM30 (Vitharana et al., 2019).

#### 2.1.2 Specified Dynamics Version of WACCMX

The Whole Atmosphere Community Climate Model with thermosphere and ionosphere extension (WACCMX) is a comprehensive numerical model, spanning the range of altitude from the earth's surface to the upper thermosphere (Liu et al., 2010, 2018; Marsh et al., 2013). The scientific goals of the model include studying the solar impact on the earth's atmosphere, couplings between atmosphere layers through chemical, physical, and dynamical processes, and the implications of the coupling for the climate and the near‐space environment. A continuous run from 2002 to 2018 of the Specified Dynamics version of WACCMX (SD‐WACCMX) has been performed on Clemson University's Palmetto supercluster and the one hourly output data are used in this study. The Modern‐Era Retrospective analysis for Research and Applications version 2 (MERRA‐2; Gelaro et al., 2017) data are nudged from the surface to 60 km. A 2D sinusoidal fitting of wavenumbers from −6 to 6 and periods of 24, 12, 8, and 6 h with respect to all longitudes and 5‐day time interval is done on the SD‐WACCMX temperature field to extract the amplitude and phase of each tide for the centered day. The 5‐day window is chosen to suppress the contamination of long‐period waves (such as PWs) on tidal retrieval while maintaining the information of short‐term tidal variability. This 2D fitting is repeated by moving its window one day ahead to obtain the tidal time series for all 18 years of data. Here, the 2D fitting with a shorter time interval than 5‐day does not change the characteristics of the tidal variability on intraseasonal (MJO) timescale. In this study, two SD‐WACCMX runs are performed. The first one is the control run with the default MERRA‐2 (introduced in Section 2.2) nudging setups. In the second run, the MJO signals in the zonal and meridional winds of the MERRA‐2 data are removed first, and then used to nudge the SD‐WACCMX (Section 3.4). By removing the MJO signals in the zonal and meridional winds up to 60 km, the effects from the background wind filtering in the troposphere and stratosphere are suppressed.

#### 2.1.3 HME Fits of Temperature Tides

**Figure 2:**
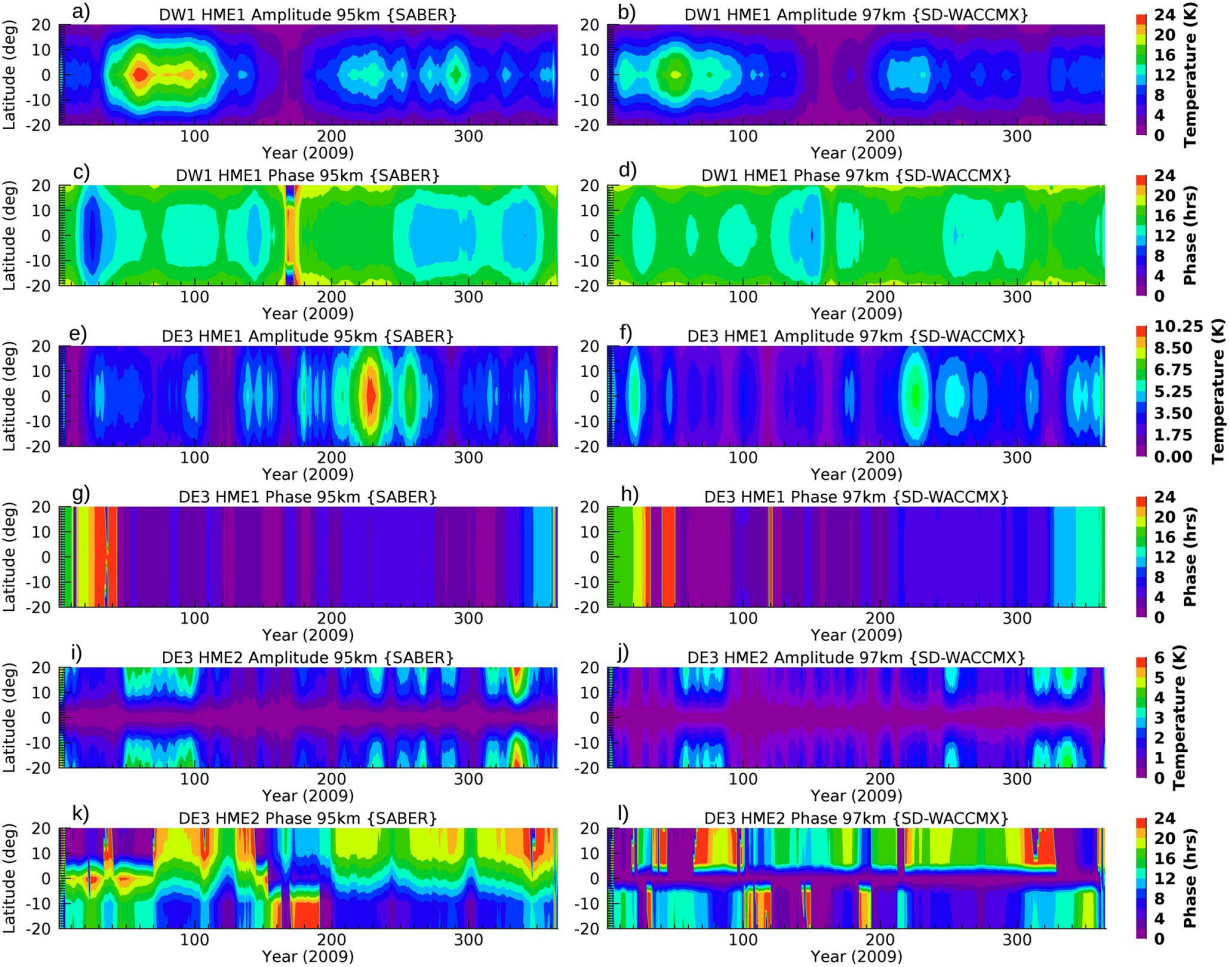
Amplitude and phase of SABER (left, 95 km) and SD‐WACCMX (right, 97 km) temperature diurnal tidal components (a–d) DW1(HME1), (e–h) DE3(HME1), and (i–l) DE3(HME2) during 2009. HME, Hough Mode Extension; SABER, Sounding of the Atmosphere using Broadband Emission Radiometry; SD‐WACCMX, Specified Dynamics version of WACCMX.

Several tidal studies in the MLT region have used HMEs fitting of tidal winds and temperature fields. This is because the latitude/height information is completely contained in the HMEs and the fit coefficients are independent of latitude and altitude and only depend on time. Classical tidal Hough functions are the orthogonal bases of the latitudinal structure of tidal components and HMEs are the altitude extension of the Hough functions including the tidal dissipation mechanisms (see Oberheide and Forbes [2008] for a detailed discussion and the numerical HME computation). Tides can be decomposed into a series of HMEs. Most of the salient features of the DE3 in the MLT can be well described by a superposition of the first symmetric (i.e., equatorial and HME1) and first antisymmetric (i.e., nonequatorial and HME2) modes (Oberheide & Forbes, 2008), while DW1 mostly consists of the first symmetric component (HME1). The symmetric DW1 mode is mainly forced by tropospheric heating, while the two dominant modes in DE3 are a consequence of tropospheric heating as well as mean zonal wind variations in the stratosphere and lower mesosphere (Oberheide & Forbes, 2008; Zhang et al., 2012). This “mode coupling” occurs because zonal mean winds determine how energy is transferred from one HME to another. The HMEs are fitted to the observations and model temperature tides in the latitude range 30°S–30°N and the height range 85–105 km. The HMEs projections of SABER (∼95 km) and SD‐WACCMX (∼97 km) temperature tides are shown in Figure 2, reconstructed using HME fit coefficients and corresponding HMEs (latitude‐altitude, as shown in Oberheide and Forbes [2008]). An 11‐day running mean smoothing is applied for plotting purposes. Note that the analysis in Section 3 uses daily tidal amplitudes and phases. The amplitude and phase comparison between SABER and SD‐WACCMX tidal diagnostics show clear agreement between observational and model studies of diurnal tides.

Figure 2 also depicts the similarity in the seasonal variation (winter/summer) and the latitude structure (symmetric/antisymmetric) of the HMEs amplitudes between SABER and SD‐WACCMX tides. DW1(HME1) and DE3(HME2) have a maximum in winter, respectively, while DE3(HME1) maximizes in summer. The seasonal characteristics of day‐to‐day variability in each HME simulated by SD‐WACCMX are consistent with the observed SABER tides. There are small differences in short‐term variations such as DW1(HME1) simulated by SD‐WACCMX compared to SABER show larger tidal amplitudes in January month. Also, SD‐WACCMX simulations of tides underestimate the short‐term variations as SABER observed tides show larger amplitudes in comparison (also shown by Pedatella et al. [2016]). Overall, these small differences may not play a large role in the intraseasonal tidal variability and in that case the model can be used to explain the observed characteristics of the tidal variability on MJO‐timescale.

Note that the seasonal variation of the DE3 HME mode strength is a combination of radiative/convective heating in the troposphere and wind filtering below the MLT region. The mode coupling in winter can also transfer wave energy from the symmetric to the antisymmetric mode (Zhang et al., 2012). Therefore, it is further necessary to perform the MJO filtering separately for the leading symmetric/antisymmetric tidal modes of each tidal component because strato/mesospheric wind filtering impacts each tidal mode differently.

### 2.2 MERRA‐2 Winds and Radiative/Latent Heating

MERRA‐2 is nudged as the lower boundary from the surface to 60 km in the SD‐WACCMX tidal simulations. We employ 3‐h output from MERRA‐2 (Gelaro et al., 2017) to obtain other parameters of interest such as winds, pressure, surface specific humidity, latent and radiative heating needed for our study. Powell (2017) demonstrates that the heating profiles (temperature tendencies [K/s or K/d] due to moist/latent and radiative processes) in MERRA‐2 are sufficiently good for MJO studies. In Section 3.3, we extract the MJO‐response in each of the tropospheric tidal forcing components, DW1 and DE3 in the latent and radiative heating profiles. In addition, we quantify the MJO‐response in MERRA‐2 tropo/stratospheric zonal and meridional wind to study the wind filtering effects on MLT tides due to the tropospheric MJO‐convection anomalies. This is to distinguish the MJO‐response in MLT tides due to the tropospheric tidal forcing from the wind filtering effect.

### 2.3 Real‐Time Multivariate (RMM) MJO Index

The RMM (Real‐time Multivariate MJO) index by Wheeler and Hendon (2004) contains a daily MJO amplitude (shown for the 2000–2019 years in Figure S1) and a phase (figure not shown) from 1974 to present which generally coincides with MJO convection locations along the equator around the globe. The MJO is characterized by large‐scale convective anomalies that develop over the tropical Indian ocean and propagate slowly (∼5 m/s) eastward over the maritime continent to the western Pacific, slowly decay over the central Pacific, and vanish over the eastern Pacific. MJO‐phases are defined as locations of MJO‐convection numbered from 1 to 8, respectively (8&1: western hemisphere and Africa, 2&3: Indian Ocean, 4&5: maritime continent, and 6&7: western Pacific). The distribution of MJO events as a function of MJO‐phases varies with seasons. Northern Hemisphere (NH) winter is the season of active MJO events (see Figure S2) when the MJO amplitude is greater than 1–1.5 for 5 consecutive days. The MJO event stays mostly in Phases 5–7 during NH winter and in Phases 1–4 during NH summer (see Figure S3 for more details where DJFM is for winter and JJAS is for summer).

## 3 Analysis and Results

### 3.1 MJO Signal Extraction in Temperature Tides: Hovmoeller Analysis

**Figure 3:**
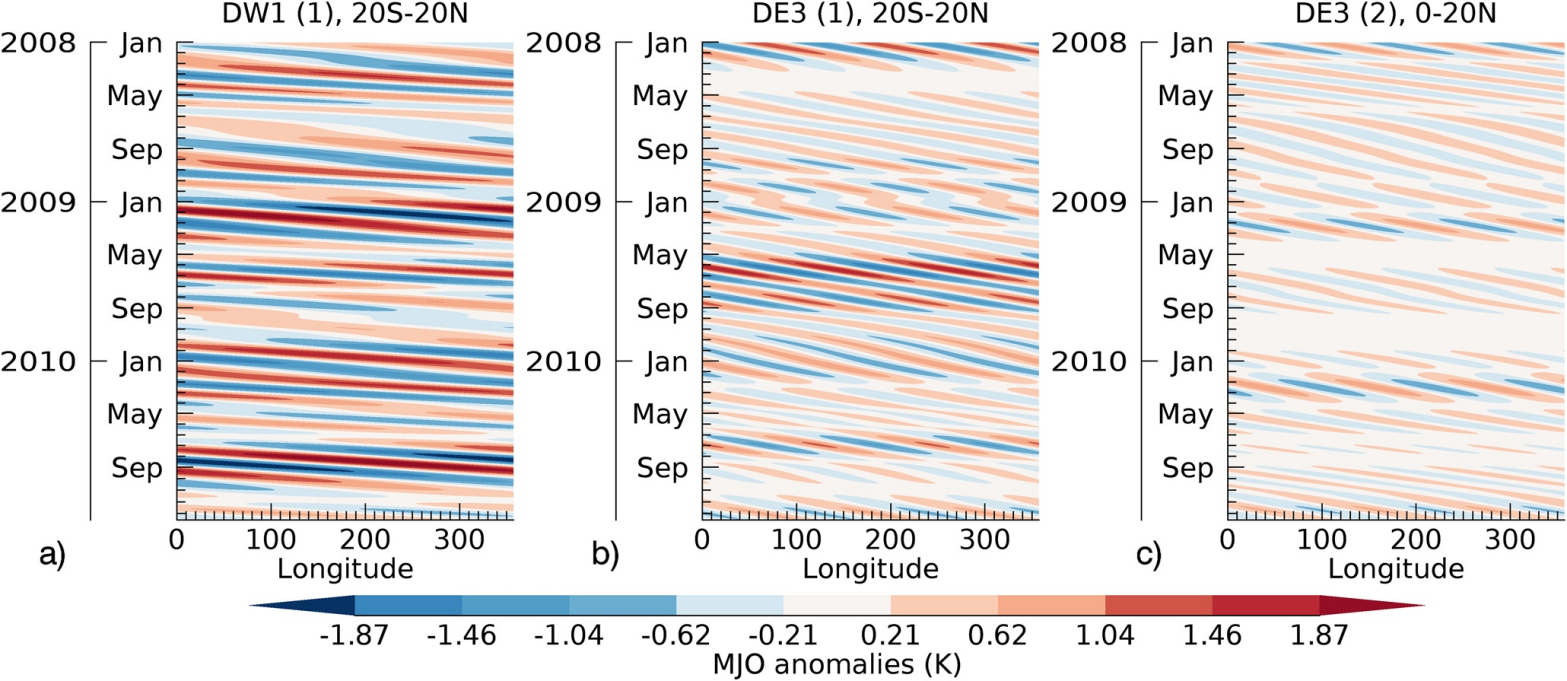
Hovmoeller plots of the SABER tidal MJO‐response during 2008–2010 and at 95 km in (a) DW1(HME1), (b) DE3(HME1), and DE3(HME2). The latitude ranges used are 20°S–20°N and 0°N–20°N for HME1 and HME2 modes, respectively. HME, Hough Mode Extension; MJO, Madden‐Julian Oscillation; SABER, Sounding of the Atmosphere using Broadband Emission Radiometry.

The SABER/MJO tidal diagnostic adopts the standard MJO diagnostic, which is Hovmoeller analysis (Wheeler & Kiladis, 1999) applied to the tidal anomalies. The steps for Hovmoeller analysis are as follows: (i) compute tidal perturbations for each day as a function of latitude and longitude (i.e., tidal deconvolution followed by HME projections explained in Section 2.1), (ii) compute a daily climatology by averaging the perturbations from multiple years (i.e., 2002–2019 for SABER), (iii) compute deviations (anomalies) from the climatology for each day, (iv) apply a 30–90 days bandpass filter, and (v) extract the eastward‐propagating signal (since the MJO is by definition eastward‐propagating). Here, the Hovmoeller analysis with tidal perturbations uses tidal phase values unlike Gasparini et al. (2020), Kumari et al. (2020), and Yang et al. (2018), which used only the tidal amplitudes (not tidal phases) for their analysis. Noise errors in the input data diminish in the filtered data because of the bandpass filtering. The last step of the Hovmoeller analysis involves latitude averaging of the tidal anomalies. Note that HME1 is symmetric with respect to the equator and HME2 is antisymmetric. Consequently, Hovmoeller analysis uses latitude averaging from 20°S to 20°N for HME1 while equator (0°) to 20°N averaging for HME2. The results of these steps are shown as Hovmoeller plots of the tidal components in Figures 3a–3c, as MJO‐signal/response at all longitudes from 2008 to 2010 of SABER tidal anomalies at 95 km in DW1(HME1) (or DW1(1)), DE3(HME1) (or DE3(1)) and DE3(HME2) (or DE3(2)), respectively.

Figure 3 shows how MJO‐response is unique for each of the HMEs. DW1(1) shows a larger response throughout the years 2008–2010 in comparison to DE3(1&2). However, the seasonal variation in MJO‐response is more evident in DE3(1&2). In particular, during 2009, DE3(2) shows a larger response in winter months and DE3(1) shows a larger response in summer months. However, the responses differ from 1 year to another year. Accordingly, it is not sufficient to study the MJO‐response characteristics in each tidal component using Hovmoeller plots as the response varies for different seasons and years. Also, as the MJO changes its locations (phases) from 1 to 8, a simple comparison of tidal MJO‐response in Figure 3 with the RMM index (Figure S1) does not provide a good measure of tidal variability with varying MJO‐locations or MJO‐phases. In the following and for further analysis, we will show how to retrieve the amplitude (or magnitude) and phase (increasing/decreasing) of the MJO‐response in tides for winter and summer seasons and as a function of MJO‐phases/locations in SABER and SD‐WACCMX tides. Note that the interannual variability of the tidal MJO‐response is not investigated in this study.

### 3.2 Statistical Characteristics of the Tidal MJO‐Response: SABER and SD‐WACCMX

**Figure 4:**
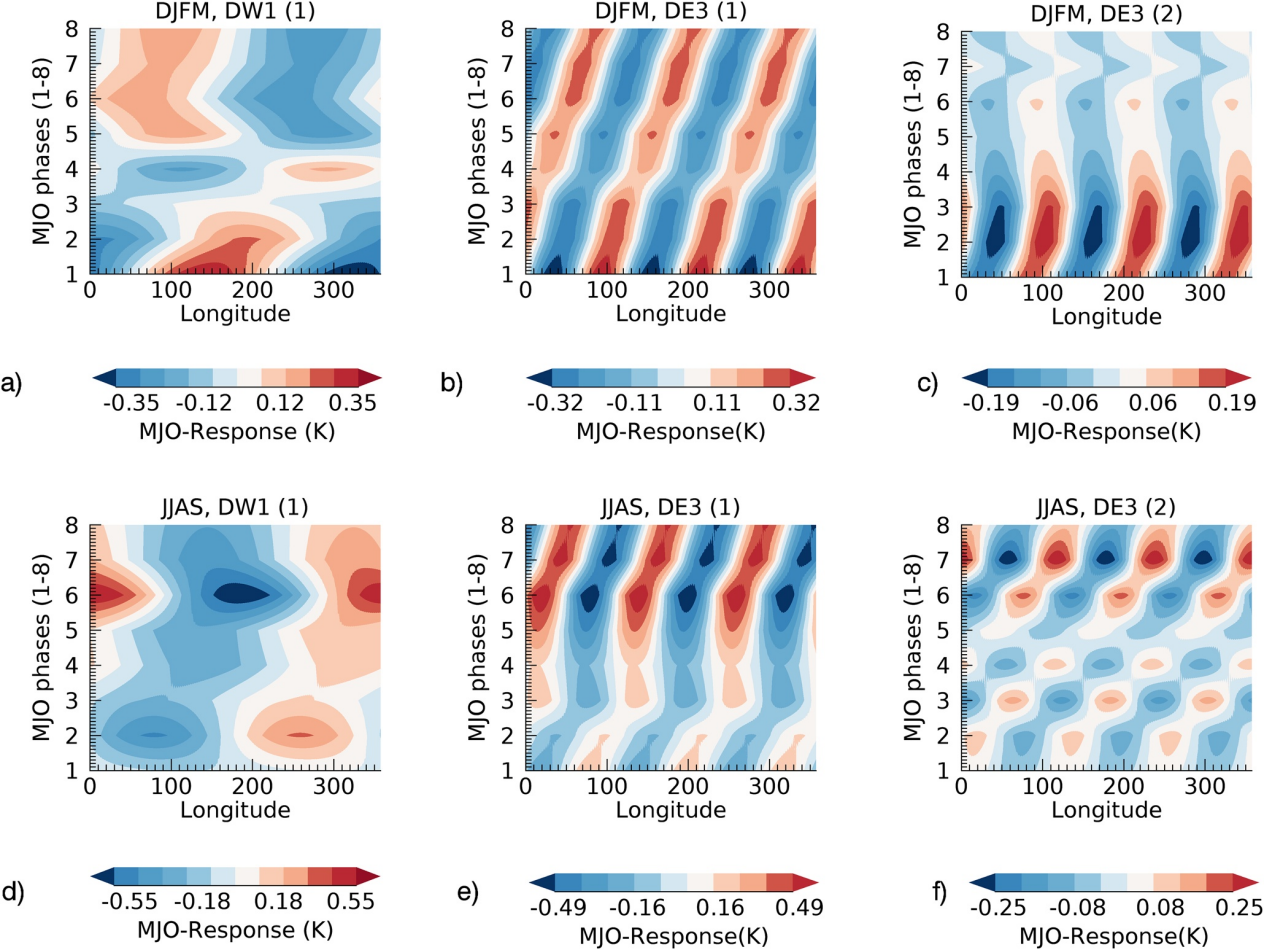
Average characteristics of the tidal MJO‐response from SABER as a function of MJO‐phases/locations, retrieved from years 2004–2017 of Hovmoeller time series of DW1(1), DE3(1), and DE3(2) in (a), (b), and (c) NH winter and in (d), (e), and (f) NH summer. The latitude averaging for HME1 modes uses the range 20°S–20°N, while the range 0°N–20°N is used for HME2 mode. HME, Hough Mode Extension; MJO, Madden‐Julian Oscillation; NH, Northern Hemisphere; SABER, Sounding of the Atmosphere using Broadband Emission Radiometry.

To establish the general consistency of the SD‐WACCMX tidal (DW1(1), DE3(1&2)) response to the MJO and SABER, we perform a statistical study as a function of MJO‐phases using a similar approach as Yang et al. (2018) and Kumari et al. (2020). Since the SD‐WACCMX study only used tides from 2002 to 2018 and the (30–90 days) bandpass filtering of the anomalies requires a buffer year at both ends, we choose the years 2004–2017 for the statistical study of the MJO‐responses in the SABER and SD‐WACCMX tides. Here, we use 2003 as a buffer year since there are no SABER data for the first few days in 2002 which helps to avoid any potential issue in the Hovmoeller analysis. To study the statistical MJO‐signal in tides with respect to the MJO‐phases in winter/summer season, we use tidal anomalies during 2004–2017 (calculated using the 2002–2019 climatology) from SABER for each of the tidal components. The first step is to identify active MJO events in each season using MJO amplitudes from the RMM index (>1–1.5; explained in Section 2.3) since we only use days corresponding to active MJO events in each season for further analysis. We then group the time series of tidal MJO‐signals (from Hovmoeller plots of 2004–2017 at all longitudes, similar to Figure 3) in eight bins corresponding to the eight MJO‐phases, using the MJO‐phase information (from the RMM index, Section 2.3) of active‐MJO days. The mean value in each bin is taken as the average/statistical measure of the MJO‐response in an MJO‐phase/bin. Figure 4 shows how the MJO‐response in the DW1 and DE3 tidal components modulates with respect to MJO‐phases 1–8 in the NH winter, that is, December‐January‐February‐March (DJFM) months and NH summer, that is, June‐July‐August‐September (JJAS) in the MLT‐region at 95 km. It is to be noted that the number of days used for the averaging in each MJO‐phase/bin varies from winter to summer. Overall, in winter, the averaging uses 307 more days for MJO‐phases 1–8 than in summer due to the MJO being more active during the NH winter (see Figure S3, JJAS and DJFM for amp > 1.5, that is, RMM amplitudes > 1.5 for at least 5 consecutive days). Also, Figure S3 shows that the active MJO events in winter have a greater number of days in MJO‐phases 5–8, while those in summer have a greater number of days in MJO‐phases 1–4. The important point here is that each phase has enough data points for statistical measure of the MJO‐response in a given season. As shown in Figure S3, we have at least 10 data points in each MJO‐phase for both MJO active conditions (amp > 1 & amp > 1.5). The lowest is 12 during JJAS in MJO‐phase 6 for the amp > 1.5 (MJO‐active) condition. The amp > 1.5 (MJO‐active) condition is a stricter condition for the selection of active‐MJO days for the statistical analysis than the amp > 1 condition. For Figure 4, we have used the stricter amp > 1.5 condition as it helps to suppress interannual variability in the MLT tidal MJO‐response. Nonetheless, we have similar proportions of days in each bin for both conditions (i.e., amp > 1 & amp > 1.5) which means that both conditions can be used to select active‐MJO days for the statistical analysis. Figure 4 shows the largest statistical response in DW1(1) (Figures 4a and 4d) with respect to the MJO‐phases, which is comparable to the MJO‐response in DE3(1) (Figures 4b and 4e) while DE3(2) (Figures 4c and 4f) has comparatively smaller response. The longitudinal structure (*x*‐axis, Figure 4) for MJO‐response in DW1(1) has zonal wavenumber 1 corresponding to DW1. Similarly, MJO‐response in DE3(1&2) shows zonal wavenumber 3 longitudinal structure corresponding to DE3. The variation of the tidal MJO‐response with respect to the MJO‐phases (*y*‐axis, Figure 4) is evident in all three tidal components.

**Figure 5:**
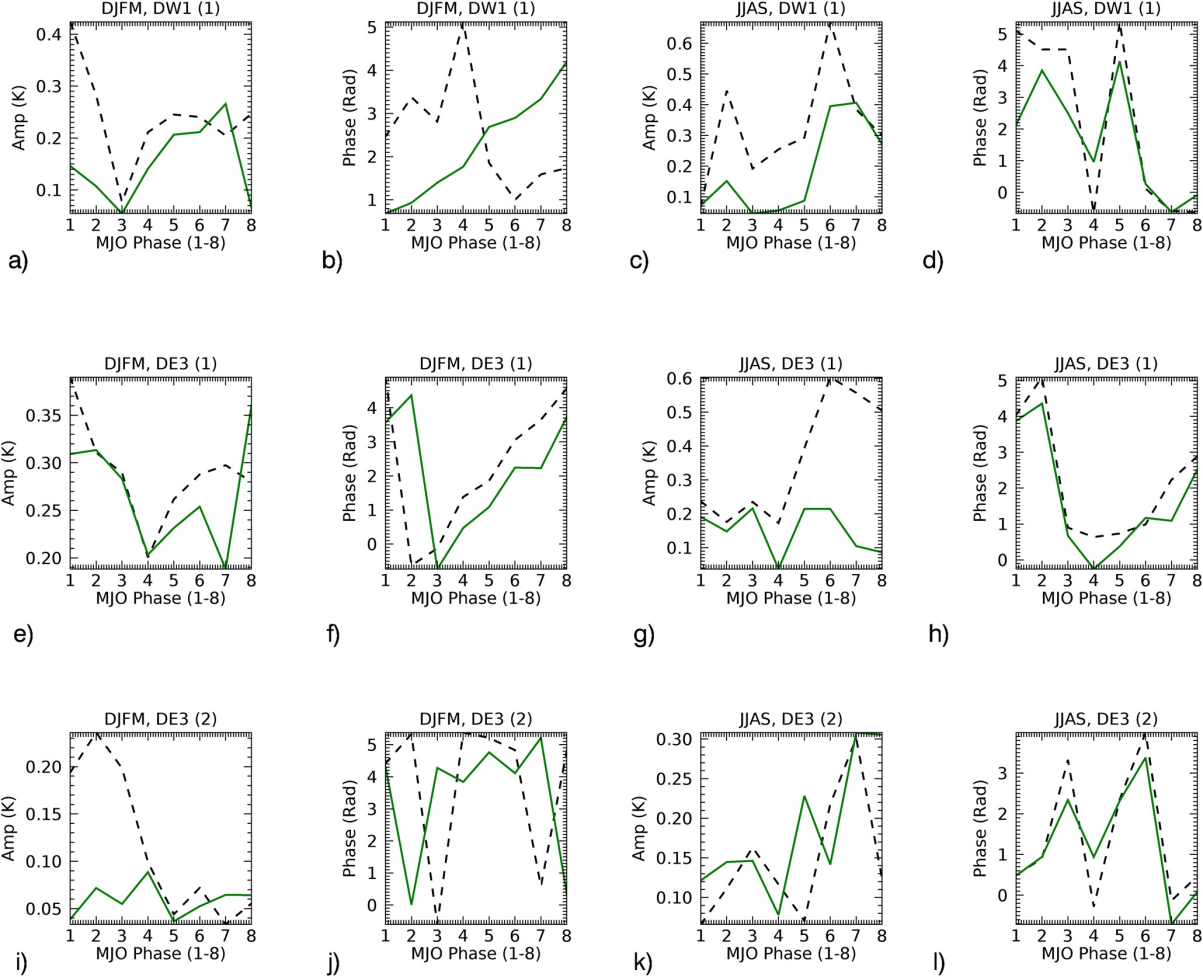
Comparative analysis of the MJO‐response in SABER tides at 95 km (black‐dashed) versus SD‐WACCMX tides at 97 km (green) tides (tidal amplitude and phases shown in Figure 2). The amplitudes and phases in longitudes are retrieved from Figure 4, where the *y*‐axis in Figure 4 is the same as the *x*‐axis here. Note that the *y*‐axis range is different for each of the plots and the difference shows the comparison in winter/summer amplitudes and phases for all three tidal components. MJO, Madden‐Julian Oscillation; SABER, Sounding of the Atmosphere using Broadband Emission Radiometry; SD‐WACCMX, Specified Dynamics version of WACCMX.

The amplitude (*A*) and phase (*ϕ*) of the MJO‐responses from Figures 4a–4f can be retrieved by fitting Acos(*nλ−ϕ*), where *λ* = longitude and *n* = 1(DW1) and 3(DE3). The statistical characteristics can then be studied in terms of the amplitude (*A*) and phase (*ϕ*) of the MJO‐response and their modulations with respect to the eight MJO‐phases. Note that the phase (*ϕ*) of the tidal MJO‐response is the phase in longitudes (i.e., *x*‐axis in Figure 4) at each of the MJO‐phases (i.e., *y*‐axis in Figure 4), where the MJO‐phase is the location of MJO‐convection given as an integer from 1 to 8. The black lines in Figure 5 show amplitudes and phases of the MJO‐response in DW1(1) and DE3(1&2) at 95 km in winter (DJFM) and summer (JJAS) season retrieved from Figure 4. The altitude structure of the amplitude of the MJO‐response in SABER as well as in SD‐WACCMX tides is shown in Figures S4 and S5, respectively. Figure 5 mainly shows the MJO‐response in temperature tides from SABER (∼95 km, black lines) as well as SD‐WACCMX (∼97 km, green lines) for NH winter (DJFM) and NH summer (JJAS) seasons.

Regarding SABER MLT tides, the noise errors are diminished in the bandpass filtering, and Kumari et al. (2020) showed that the error due to the tidal deconvolution and HME fittings (which is ∼1K) constitutes 1%–9% (0.02–0.12K) error in intraseasonal signal in tidal amplitudes (anomalies) varying as a function of MJO‐phases. Despite the error, the modulation of intraseasonal tidal amplitudes with respect to the MJO phases was found to be significant and hence the MJO‐response in SABER tides using Hovmoeller analysis can be considered statistically significant. An error estimation of line plots in Figure 5 using Hovmoeller analysis will give misleading information as the positive and negative anomalies (depending on different longitudes) are being averaged. This is due to the fact that the tidal phases are included in the Hovmoeller analysis.

We now discuss the comparative analysis of the MJO‐response from Figure 5 in each of the tidal components at 95 km from SABER (black) observations and at 97 km from SD‐WACCMX (green) simulations. The important insight is derived by comparing the characteristics of modulating amplitudes/magnitude and phase of the response at different MJO locations. The magnitude of MJO‐response in SABER diurnal tides (black curves in Figures 5a–5e, 5g, 5i, and 5k) shows two peak structures as a function of MJO‐phases/locations (1–4 & 5–7; *x*‐axis in Figure 5) which is consistent with the SD‐WACCMX MLT tidal response except for DE3 (HME2) (Figure 5k) at MJO‐location 5 during summer. Here, consistency is referred to when the modulations (increasing/decreasing) of the magnitude of MJO‐response with different MJO‐locations between SABER and SDWACCMX tides are similar in most cases. Note that SD‐WACCMX tides at 95 km are underestimated in comparison to SABER tides (Figure 2) and thus the magnitude or amplitudes of the response in SD‐WACCMX tides are smaller than that of SABER. The seasonal variation in the magnitude (Figure 5) of the double‐peak structure in SABER MLT tides (black curves) is more evident than in SD‐WACCMX tides (green curves) as a function of MJO‐phases (1–4 & 5–7).

The higher magnitude of the response occurs at MJO‐locations 1–2 during winter while at MJO‐locations 6–7 during summer. On another note, the phases of the MJO‐response in SABER diurnal tides (black curves in Figures 5b–5f, 5h, 5j, and 5l) during winter/summer season are generally consistent with the SD‐WACCMX diurnal tides (green curves) with the exception of DW1 (HME1) and DE3 (HME2) during winter, as shown in Figures 5b and 5j. Some systematic phase differences can be caused by the fact that the tides in the model and observations have a different vertical wavelength (e.g., SD‐WACCMX tides maximize at slightly higher altitudes than SABER tides). Also, on another note, if the model simulations considered the nonlinear mechanisms through which the MJO can be imprinted on tides, we can expect to have a better agreement between MJO‐responses characteristics in SABER and SD‐WACCMX tides.

Note that there is considerable variation taking place as a function of MJO‐phases from winter to summer in all three tidal components and the amplitude of the MJO‐response is larger in summer than winter. This can also be seen in Figure 4. In Figure 4, the contour values vary in the range of ±0.35, ±0.32, and ±0.19 during winter which is smaller than the values during summer, that is, ±0.55, ±0.49, and ±0.25 for DW1(1), DE3(1), and DE3(2), respectively. Similarly, Table 1 shows the measure of total MLT tidal variability due to the tropospheric MJO by measuring peak‐to‐peak (maxima‐minima) difference in the amplitudes of the MJO‐responses from Figure 5, which shows that higher differences occurred during summer than winter. This is surprising as the MJO is mainly active during winter season.

**Table 1:** The Peak‐to‐Peak Difference in Kelvins From Figure 5 at all MJO‐Locations for the Magnitude of the SABER MLT Tidal MJO‐Response (Black Lines) in Winter and Summer Seasons

Tidal components	Winter (DJFM)	Summer (JJAS)
DW1(HME1)	0.35	0.60
DE3(HME1)	0.19	0.43
DE3(HME2)	0.20	0.24

Abbreviations: DJFM, December‐January‐February‐March; JJAS, June‐July‐August‐September; MJO, Madden‐Julian Oscillation; MLT, mesosphere/lower thermosphere; SABER, Sounding of the Atmosphere using Broadband Emission Radiometry.

### 3.3 MJO‐Response to Tidal Forcing in the Troposphere

Tropospheric forcing needs to be studied using the same frequency/wavenumber picture as in the MLT (e.g., DE3 and DW1) and subsequently their Hough mode (HM, not HME) projections. We first calculate the DE3 and DW1 forcing in radiative and latent heating (i.e., tidal forcing in the troposphere region) from MERRA‐2 (2002–2019, in Kelvin per day) and then perform HM projection after averaging the forcing between 100 and 500 hPa (integrating the heating altitudes) in the troposphere. The retrieval of MJO‐response in both radiative and latent forcing is derived using Hovmoeller analysis, just as in Section 3.1 (i.e., for SABER and SD‐WACCMX tides in the MLT region).

**Figure 6:**
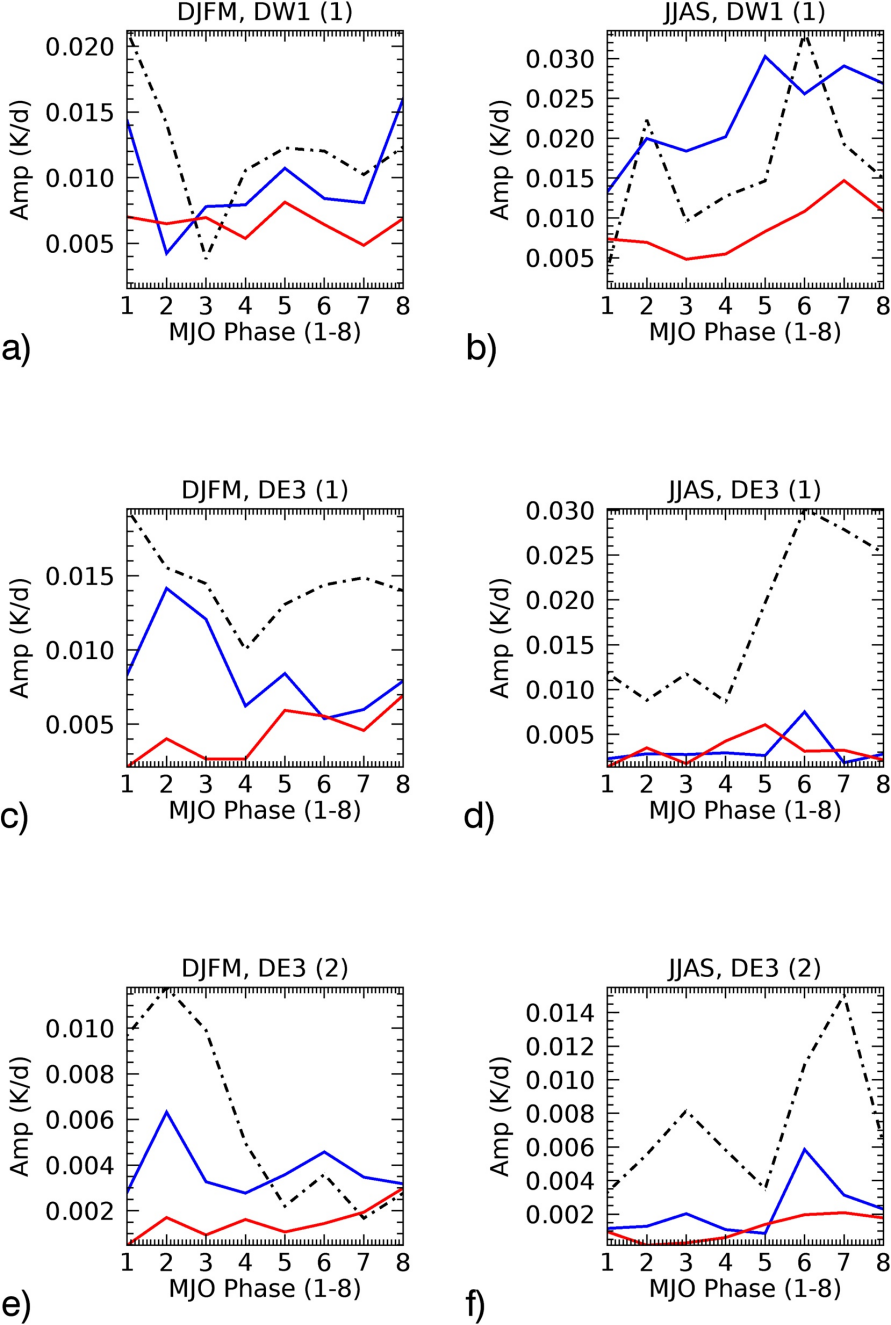
Comparative analysis of the magnitude of the MJO‐response in SABER (black‐dotted) with the response in the radiative heating (red) and latent heating (blue). The unit of radiative and latent heating forcing is K/day, while SABER tides (95 km) are measured as temperatures. The MJO‐response in SABER tides is multiplied by 0.05 and the response in latent heating is multiplied by 3 for plotting purposes. MJO, Madden‐Julian Oscillation; SABER, Sounding of the Atmosphere using Broadband Emission Radiometry.

Figure 6 shows the amplitudes (*A*) of the MJO‐response in radiative (red lines) and latent heating (blue lines, the amplitudes shown as three times the latent heating for plotting purposes) in each season for each of the tidal components. The phases (*ϕ*) of MJO‐response for tidal heating are shown in Figure S6. The black lines in Figures 6 and S6 indicate amplitudes and phases of the MJO‐response in SABER MLT‐tides. Note that the SABER amplitudes are in Kelvin and multiplied by 0.05 for plotting purposes. The SABER MLT‐tidal MJO‐response uses the same MJO‐active condition (RMM amplitudes > 1.5) as Section 3.2. The black dotted lines in Figure S6 are same as the corresponding black dotted lines in Figure 5, while the negative phase values of MJO‐response (Figures 6a–6f) have not been shifted to positive values by adding 2π with respect to the corresponding phases in Figure 5. This is for plotting purposes and to do the comparative analysis with the phases of MJO‐response in radiative and latent heating.

Here, the active MJO events for the tidal heatings (i.e., red and blue lines in Figures 6 and S6) are chosen as the days when MJO amplitudes are greater than 1 for 5 consecutive days, which is a less strict condition to select active MJO events compared to the 2004–2017 analysis from Section 3.2 (which used MJO amplitudes greater than 1.5 for 5 consecutive days). This is for plotting purpose because the MJO‐response retrieved using this active‐MJO condition provides better agreement with SABER MLT‐tidal MJO‐response than the condition used in Section 3.2. As described in Section 3.2, Figures S2 and S3 show that active MJO events have similar proportions of days in each bin for both conditions (i.e., RMM amplitudes > 1 and > 1.5), so choosing either of the conditions should not change the characteristics of the MJO‐response as a function of MJO‐phases. For RMM > 1.5 condition, Figure S7 shows the heating amplitudes and phases in comparison with SABER tides. As expected, the phases still look consistent in Figures S6 and S7, but the amplitude characteristics may vary between Figures 6 and S7. This is because the interannual variability in two different MJO active conditions is being mapped differently between Figure 6 (2004–2017) and the amplitude line plots of Figure S7 (2008–2010).

From Figure 6 during summer (JJAS), the characteristics of amplitude modulation with MJO‐phases/locations in latent tidal tropospheric heating (blue lines) are consistent with the amplitude modulation in SABER (black) except for DE3(1) during winter. The “consistent” is referred to whether there is an enhancement in forcing when there is an enhancement of tidal MJO‐response. This can also be understood from Table 2 which also shows that the response in radiative heating is somewhat consistent with the MLT DW1(1) tidal response during summer and winter. This is expected as radiative heating is the main forcing factor for DW1 while latent heating is important for DE3 forcing.

**Table 2:** The Table Shows Whether the Modulation of Magnitude of the MJO‐Response With MJO‐Locations in the Radiative and Latent Tidal Heating From Figure 6 is Consistent With the MLT Tidal Response From SABER or Not

Tidal components	Winter (DJFM)		Summer (JJAS)	
	Radiative	Latent	Radiative	Latent
DW1(HME1)	Yes	Yes	Yes	Yes
DE3(HME1)	No	No	No	Yes
DE3(HME2)	No	Yes	No	Yes

Abbreviations: DJFM, December‐January‐February‐March; JJAS, June‐July‐August‐September; MJO, Madden‐Julian Oscillation; MLT, mesosphere/lower thermosphere; SABER, Sounding of the Atmosphere using Broadband Emission Radiometry.

The phases of MJO‐response in either season in Figure S6 are not consistent between the tidal forcing and SABER MLT tides (black dotted curve) except for DE3(1) with latent heating (blue) during winter season. This is, however, expected as wind filtering and additional effects are also important in transmitting the MJO signal into the tides, as discussed more closely in the following section. Interestingly, for DW1(1) and DE3(2), the modulation of the phases of the MJO‐response with MJO‐phases in radiative heating (red lines in Figure S6, heating altitudes 100–500 hPa integrated) is generally consistent with the phases of the MJO‐response in latent heating (blue lines in Figure S6), but the scenario is different for the DE3(1) during winter and DE3(2) during summer. Yang et al. (2018) discussed that increased moisture due to latent heating forcing in troposphere results in an enhanced radiative heating at the MJO‐phases (i.e., location of MJO‐convection). In other words, Yang et al. (2018) expected that the latent and radiative MJO‐forcing to tides are related. Understanding the apparent phase inconsistencies of MJO‐response among radiative and latent forcing for DE3(1) while being consistent for DE3(2) requires additional insights and is beyond the scope of this study. Nonetheless, the latent and radiative MJO‐forcing together explain important characteristics of amplitudes (magnitude) of MJO‐response as a function of MJO‐phases in the MLT region. The amplitudes of MJO‐forcing in the latent and radiative tidal heating terms are comparable in the winter and summer seasons (Figure 6), which does not explain the seasonal difference in amplitudes of the MJO‐response (function of MJO‐phases) in SABER and SD‐WACCMX tides (Figure 5).

### 3.4 Delineating Wind Filtering Effect in Tropo/Stratospheric Zonal and Meridional Winds

**Figure 7:**
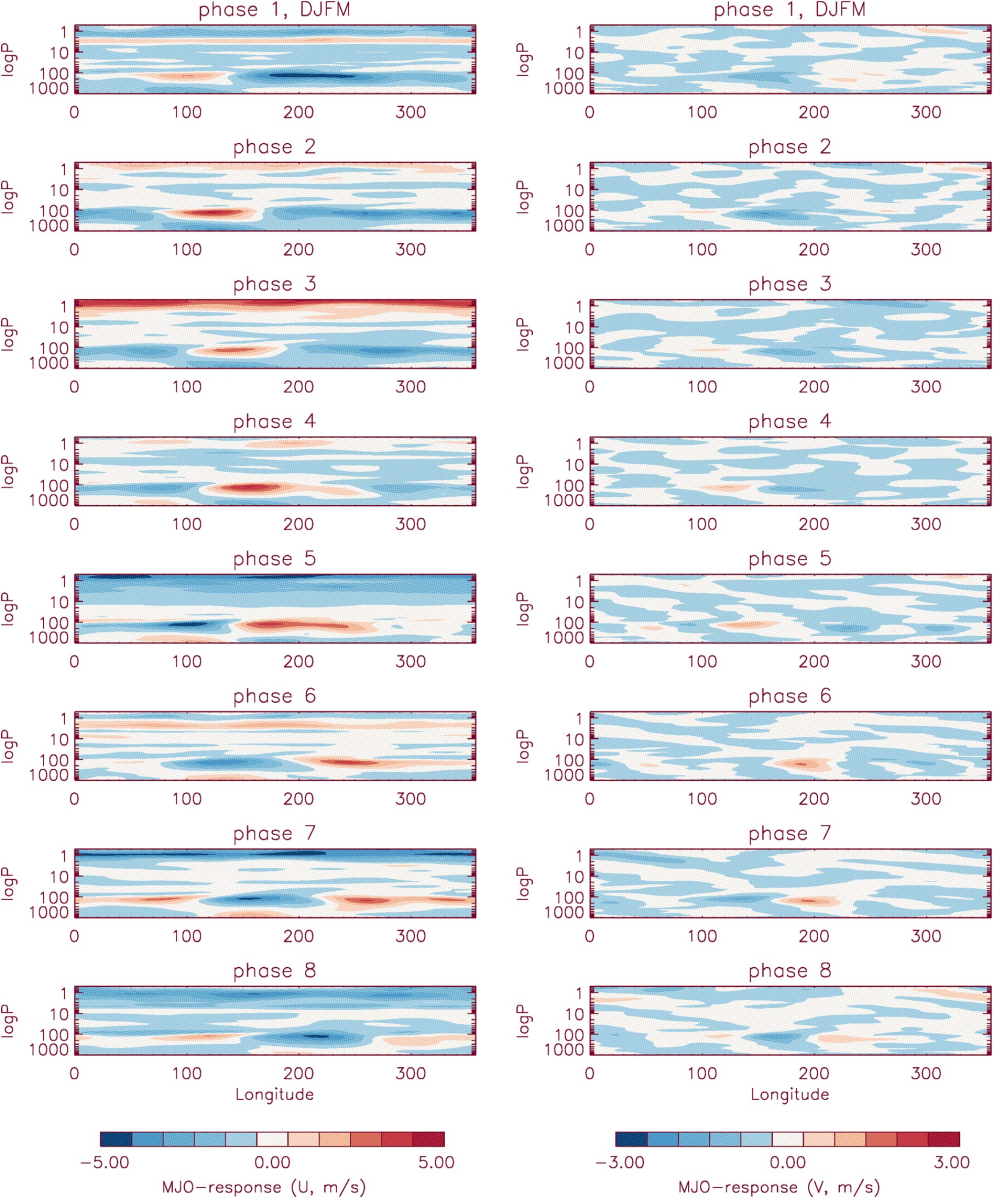
Longitudinal structures of the MJO‐response in MERRA2 zonal (*U*, left in the figure) and meridional (*V*, right in the figure) winds at the stratosphere and troposphere altitudes are shown at each of the MJO‐phases/locations. The months used are December–March for years 2008–2010 and the latitudes are averaged between 0°S and 20°S. MJO, Madden‐Julian Oscillation.

To extract the wind filtering effect in the tropo/stratosphere, the MJO‐response in zonal (*U*) and meridional (*V*) winds from MERRA‐2 are analyzed. This analysis uses the years from 2008 to 2010, which are characterized by several large MJO‐activities in the tropical convection, the so‐called “year of tropical convection” (Waliser et al., 2012). Previously, the MJO‐response to tropo/stratospheric zonal winds in different MJO‐phases has been discussed by Alexander et al. (2018) using another methodology, which used the difference between MERRA‐2 zonal wind composites in calculated each MJO‐phase for active MJO days (with amp > 1 condition) and the MERRA‐2 wind 2003–2011 climatology (monthly mean zonal wind) weighted by the frequency of occurrence in that month in each MJO phase but without selecting the active MJO events. Essentially, Alexander et al. (2018) calculated the MJO‐anomalies using the climatology per MJO‐phase and by comparing with and without the active MJO events. Our analysis first calculates the anomalies and then uses MJO‐phase information for each active MJO‐events to get the variability characteristics as a function of MJO‐phases. Here, we use the Hovmoeller analysis to extract the eastward propagating MJO‐response in zonal and meridional wind anomalies from troposphere to stratosphere (1000–0.5 hPa). The climatology was calculated using 18 years (2002–2019, same number of years as used in Section 3.1) of MERRA2 winds to get the wind anomalies of 2008–2010. Figure 7 shows the longitudinal variability of the MJO‐response during 2008–2010 in zonal (left) and meridional (right) wind anomalies for winter season (i.e., DJFM) in each of the MJO‐phases. One can see the eastward propagating longitudinally dependent MJO‐response evident in the troposphere (i.e., >120 hPa) for both zonal and meridional winds, while longitudinally independent zonal wind anomalies can be seen in the stratosphere (30–0.5 hPa). The MJO‐response in stratosphere zonal wind anomalies changes its sign depending on the MJO‐phase. Note that the wind anomalies in zonal winds are large (±5 m/s) compared to those in meridional winds (±3 m/s). An analysis for summer months shows a bigger MJO‐response in zonal winds up to ±8 m/s, while the response in meridional winds is of similar magnitude (±3 m/s) (figure not shown). Most importantly, for winter months, the zonal wind anomaly variation with MJO‐phases is consistent with the findings by Alexander et al. (2018), except that the magnitude of the variations in their study is an overestimate of up to ±5 m/s when compared to our findings. This could be due to the difference in analysis methods as Alexander et al. (2018) used a weighting factor for each MJO‐locations to calculate the climatology which possibly contributed to the larger anomalies.

We use the information in Figure 7 to further extract the tropo/stratospheric wind filtering effect from the MJO‐response in the MLT region tides**.** After the removal of the MJO signals in the horizontal winds (*U* and *V*) of MERRA‐2 data, another SD‐WACCMX run with the same configuration as the previously mentioned run (Section 2.1.2) but using MERRA‐2 winds without the MJO is performed from 2008 to 2010. The same 2D fitting process is carried out to extract the temperature tides from this modified model run. For the comparative study of the tidal anomalies, the Hovmoeller analysis uses the same climatology as used in the analysis with the first (unmodified) SD‐WACCMX run. The amplitude and phase of the MJO‐response in each of the tidal components were obtained with the modified model run using the same methodology discussed above, but the years used for the statistical measure of the MJO‐response are 2008–2010. Here, the active‐MJO days were chosen with the less strict condition (i.e., amp > 1 as discussed in Section 3.3; this is to provide enough data points for the statistical measure of MJO‐response).

**Figure 8:**
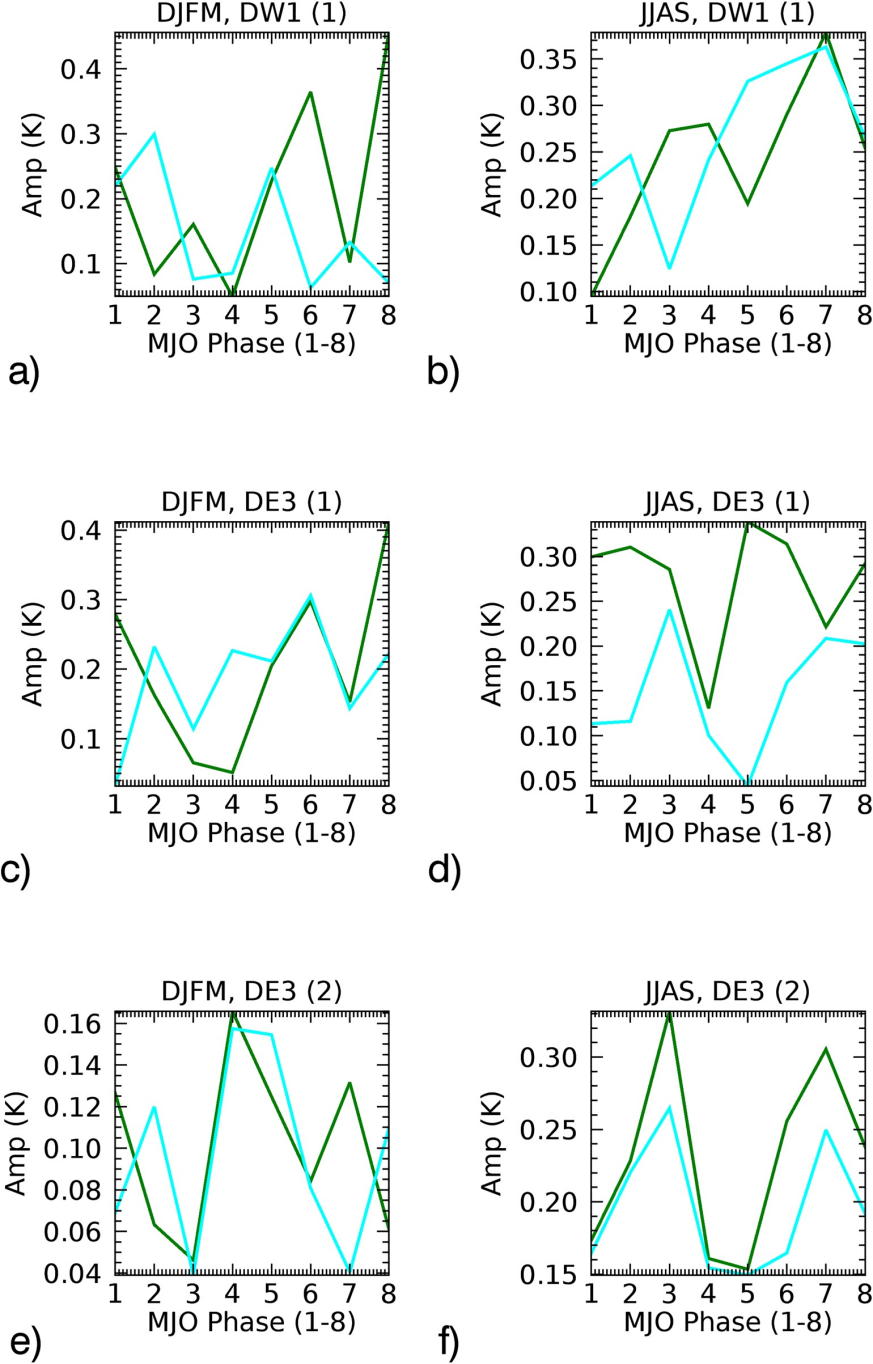
Same as Figure 6 but the comparison shown is between the two SD‐WACCMX model runs using 2008–2010 data. The green lines are for the first model run with MJO while the cyan lines are for the second model run without MJO in winds. MJO, Madden‐Julian Oscillation.

The amplitudes of the MJO‐responses in the modified SD‐WACCMX MLT‐tides are shown in Figure 8 in cyan‐color lines along with the MJO‐response in the green lines of the unmodified (wind filtering effect not removed) SD‐WACCMX MLT‐tides. The corresponding phases of MJO‐response for both simulations are shown in Figure S8. Note that the analysis for the first model run is done using the years 2008–2010 for Figure 8, so the green line plots in Figure 8 are not same as the green line in amplitude plots of Figure 5, which represent the statistical MJO‐response for the years 2004–2017. This is mainly due to interannual variability in the MJO‐responses in MLT‐tides. The phases shown in Figure S8 in both seasons for all the tidal components are consistent with the unmodified model runs except for DE3(HME1). The peak‐to‐peak difference in amplitudes of the MJO‐responses (from Figure 8) also has small differences from the unmodified model runs. The only exception is that the MJO‐response in DE3 seems to decrease significantly in HME1 (Figure 8d) and moderately in HME2 (Figure 8f) without the wind filtering effect in summer. This could be due to the larger MJO‐response in the stratospheric zonal winds during summer months (i.e., larger wind‐filtering effect to tides) contributing to the MLT‐tidal response, as discussed above using MERRA‐2 winds. The changes in characteristics of amplitudes as a function of MJO‐locations can also be noticed in Figure 8, the detailed study of such changes is out of the scope of this study. It is surprising that there is not a significant phase difference between the two model runs. Nonetheless, from Figure 6, tidal heating can explain the average characteristics of tidal MJO‐response in the MLT region. Overall, the wind‐filtering effect in the tropo/stratosphere is not the dominant mechanism by which the MJO‐response is mapped into the MLT‐tides. Therefore, tropospheric radiative and latent tidal forcing in MERRA2 makes the most significant contributions to the process by which the MJO response is imprinted on the tides in the MLT.

## 4 MJO‐Response in the Tidal Momentum Budget in the MLT Region

To understand the underlying physical mechanisms responsible for transmitting the MJO‐response in the MLT tides, we investigate the tidal momentum budget from SD‐WACCMX above the stratosphere up to the MLT region in response to the MJO. Note that MERRA‐2 with the MJO included is used for nudging in SD‐WACCMX simulations of tides below ∼60 km. For each MJO‐phase and season group, we diagnose the MJO‐signal in classical, advection, and GW terms in the zonal and meridional tidal momentum equations:

(1) $$\frac{\partial u}{\partial t}=fv-\frac{1}{a\;\cos\;\phi }\frac{\partial \Phi }{\partial \lambda }-\left(\frac{u}{a\;\cos\;\phi }\frac{\partial u}{\partial \lambda }+\frac{v}{a}\frac{\partial u}{\partial \phi }\right)+\frac{uv}{a}\tan\;\phi +F_{\text{GW}x}+X$$

(2) $$\frac{\partial v}{\partial t}=-fu-\frac{1}{a}\frac{\partial \Phi }{\partial \phi }-\left(\frac{u}{a\;\cos\;\phi }\frac{\partial v}{\partial \lambda }+\frac{v}{a}\frac{\partial v}{\partial \phi }\right)-\frac{u^{2}}{a}\tan\;\phi +F_{\text{GW}y}+Y$$

The first two terms on the right‐hand side of the equations are the classical tidal terms due to Coriolis force and pressure gradient force. The other terms are nonclassical and describe advection, curvature and GW forcing. *X* and *Y* represent the remaining nonconservative mechanical forcing. Following Lu et al. (2012), we use the SD‐WACCMX data to quantify the relative strengths of the MJO signal in each term of Equations 1 and 2 for the tidal components DW1 and DE3. The tendency term, Coriolis term, pressure gradient term, advection term, curvature term, and GW forcing term are first calculated using a centered difference scheme based on the diagnostic outputs of winds, geopotential height, and GW forcing. Then each term is decomposed into tidal components by applying the same 2D fitting method as we used for temperature. By conducting the study as a function of MJO phase and season, we determine how individual terms in the tidal momentum budget respond to the MJO in different phases and seasons, and how they contribute to the overall MJO in the MLT tides.

**Figure 9:**
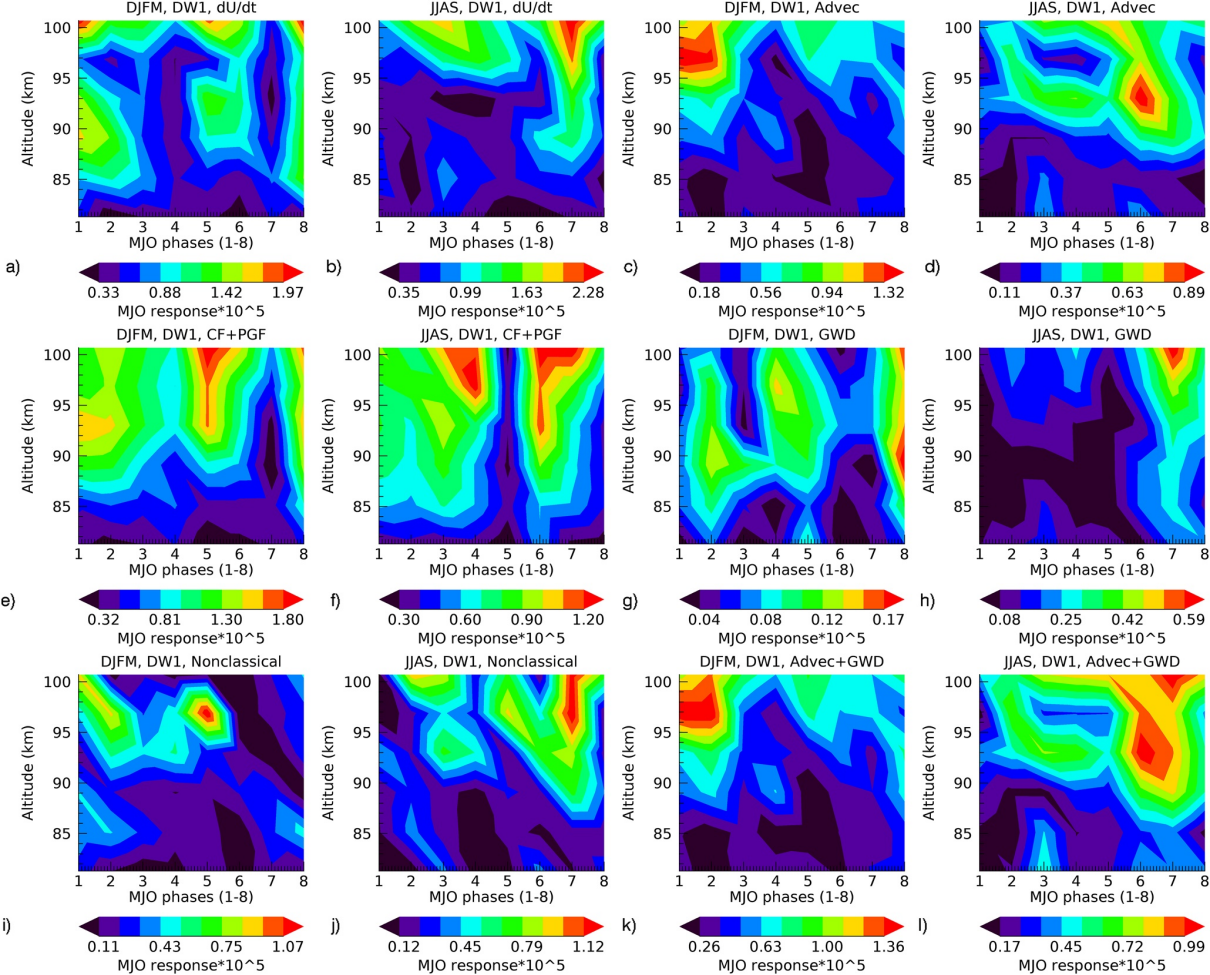
Amplitude of the MJO‐response in the zonal wind momentum forcing for DW1(1) tidal component in both winter (DJFM) and summer (JJAS) seasons, where the MJO‐response is in units of 10^−5^ m/s/s for each of the contour plot and the ranges of response (i.e., color bars) for each term are different. Nonclassical terms (i) & (j) represent the difference in (a) & (b) zonal wind tendencies (dU/dt) and (e) & (f) classical terms (Coriolis force + pressure gradient force), respectively. The individual nonclassical terms are the advection (c) & (d) and GWDs (g) & (h). Advection and GWD together at MLT altitudes are shown in (k) and l). DJFM, December‐January‐February‐March; GWD, gravity wave drag; JJAS, June‐July‐August‐September; MJO, Madden‐Julian Oscillation.

We study the MJO‐response diagnostics (i.e., Hovmoeller analysis) on both the zonal and meridional momentum budget corresponding to each of the tidal components. For DW1 zonal and meridional winds, the first dominant mode is the antisymmetric (Hough) mode, while the pair of first and second dominant modes for DE3 zonal winds are the symmetric and antisymmetric modes, and for DE3 meridional winds, these are the antisymmetric and symmetric modes, respectively. This corresponds to the MJO‐response diagnostics in the tidal DW1‐HME1, DE3‐HME1, and DE3‐HME2 components in temperature. Here, the MJO‐response diagnostics of the individual terms in the momentum budget does not use the HME fitting as there are no HMEs for the tidal momentum equation individual terms. At present, symmetric and antisymmetric modes in each individual term are computed by averaging the MJO‐response anomalies (after Hovmoeller analysis) between ±25° latitude as even and odd functions (5°S–25°S for odd modes while 15°N–15°S for even modes). Figure 9 exemplifies the amplitude (phases not shown) of the MJO‐response in the individual terms in the DW1 zonal wind momentum budget in the MLT region (80–100 km) as a function of MLT altitudes and MJO‐phases. The peak‐to‐peak differences from Figure 9 are shown in Table 3 as a measure of total contribution to the tidal MJO‐response due to individual classical and nonclassical terms in the momentum budget of MLT tides. The corresponding figure for the meridional momentum terms and figures for the DE3(1&2) zonal and meridional terms are shown in Figures S9–S13.

**Table 3:** The Peak‐to‐Peak Difference in Units of 10^−5^ m/s/s of the Magnitude of the MJO‐Response in the Individual Terms in the DW1 Zonal Wind Momentum Budget (From Figure 9) in the MLT Region (80–100 km) as a Function of MLT Altitudes and MJO‐Phases/Locations

Forcing terms in the DW1(1) zonal momentum budget	Winter (DJFM)	Summer (JJAS)
dU/dt	1.7	2.77
CF + PGF	1.63	1.75
Advec + GWD	1.38	2.53

Abbreviations: DJFM, December‐January‐February‐March; JJAS, June‐July‐August‐September; MJO, Madden‐Julian Oscillation; MLT, mesosphere/lower thermosphere; SABER, Sounding of the Atmosphere using Broadband Emission Radiometry.

Figure 9 shows that classical momentum forcing such as Coriolis and pressure gradient together (Figures 9e and 9f) represent most features of the MJO response in the zonal momentum budget (Figures 9a and 9b). Advection and GWD together in (Figures 9k and 9l) explain most features of the nonclassical momentum forcing (Figures 9i and 9j) at the MLT altitudes. Note that the advection forcing is larger than the GWD forcing. This is true for each of the tidal components, as shown in Figures S9–S13. The seasonal variation from DJFM (winter) to JJAS (summer) of the MJO‐response as a function of MJO‐phases of each of the individual term follows that of the wind tendencies, such as the enhanced response in MJO‐phases 1–4 in DJFM while in MJO‐phases 5–7 in JJAS. Table 3 shows that all the individual terms are larger (i.e., difference between maxima and minima of contour values denoted as the values on color bars in Figure 9) in JJAS than in DJFM, which is possibly the reason why there is a bigger tidal MJO‐response in JJAS, shown in Figure 5. This is interesting as the MJO is more active in winter (DJFM) season and the response in the tides is larger in summer (JJAS).

**Figure 10:**
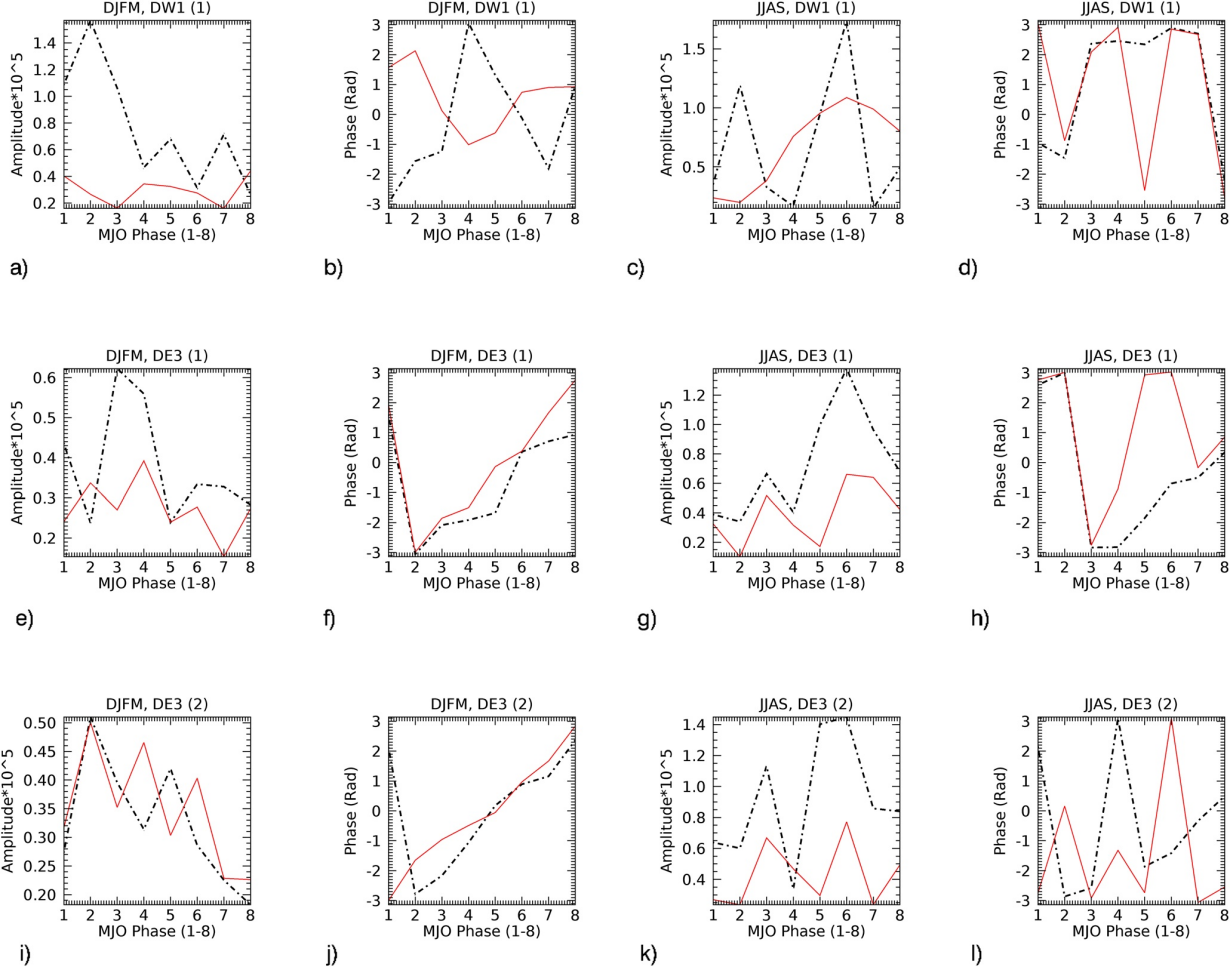
The amplitude and phase relationship between advection (dotted black lines) and GWD (red) momentum forcing to (a–d) DW1(1), (e–h) DE3(1), and (i–l) DE3(2) tidal zonal winds are shown in both winter (DJFM) and summer (JJAS) months at 97 km in the MLT region. The amplitude of MJO‐response in GWD has been multiplied by 2 for plotting purposes and the unit of amplitude is m/s/s. DJFM, December‐January‐February‐March; GWD, gravity wave drag; JJAS, June‐July‐August‐September; MLT, mesosphere/lower thermosphere; MJO, Madden‐Julian Oscillation.

**Figure 11:**
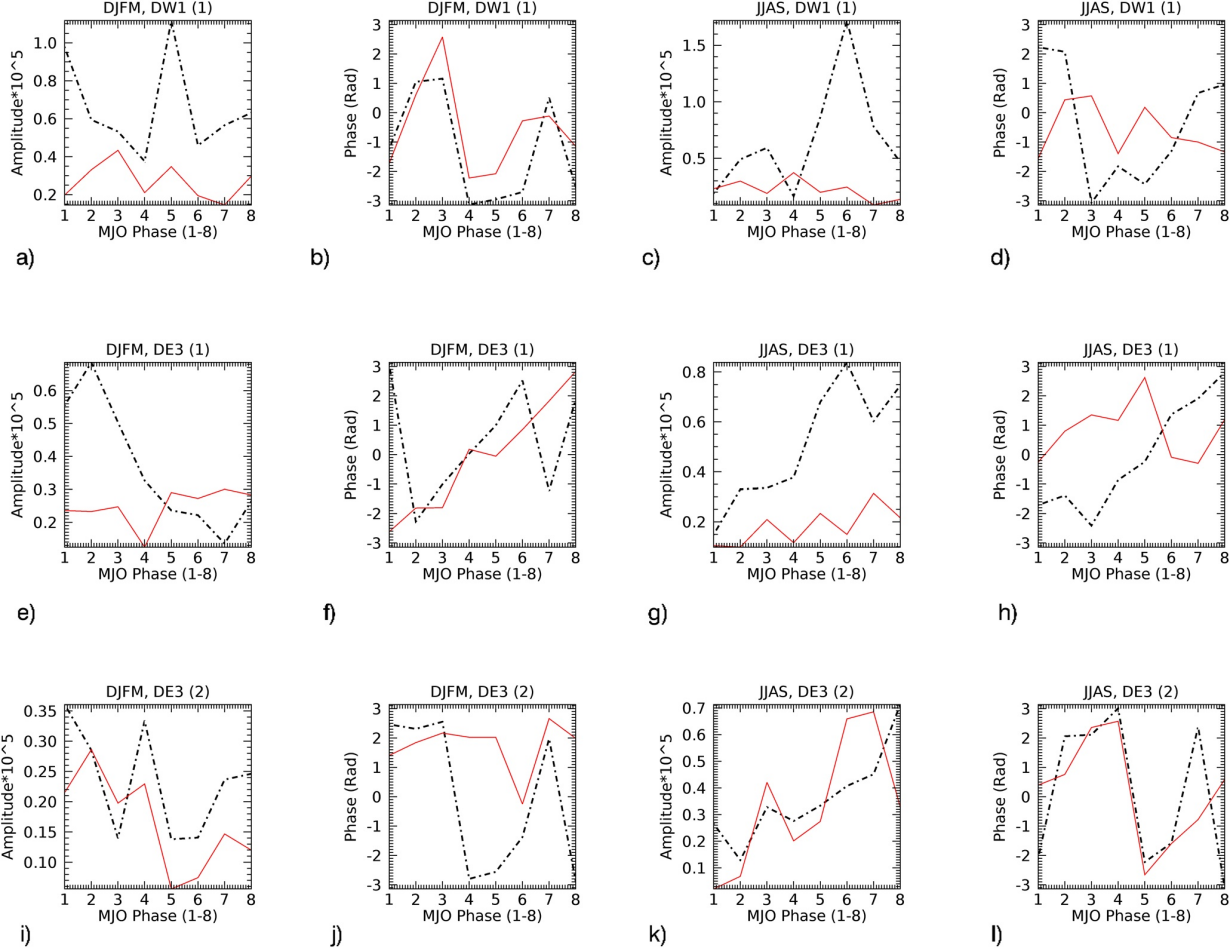
Same as Figure 10, but for the advection and GWD in meridional wind momentum budget. GWD, gravity wave drag.

Next, we discuss the role of nonclassical forcings such as advection and GWD in the zonal and meridional wind momentum budget for all three tidal components. Figures 10 and 11 show the comparative analysis between advection (dotted black lines) and GWD (red lines) momentum MJO‐forcing (or MJO‐response) in all three tidal components at 97 km (same altitude used in Figures 5 and 8) in zonal and meridional tidal winds, respectively. Basically, we perform the amplitude and phase diagnostics of the MJO‐response in advection and GWD forcing and plot them together in Figure 10 for zonal advection and zonal GWD and in Figure 11 for meridional advection and meridional GWD. The zonal advection MJO‐forcing amplitudes in DE3(1) and DE3(2) are comparable in both seasons while the meridional advection MJO‐forcing in DE3(2) decreases in winter along with GWD. Both the advection and GWD MJO‐forcing amplitudes are more significant for summer than winter, which possibly explains the smaller MJO‐response in SD‐WACCMX DE3(2) tidal component in winter than summer (Figures 5i and 5k). This is surprising since DE3(2) climatology along with the MJO‐activity maximizes in winter months. However, the MJO‐response in SABER DE3(2) is comparable in both seasons which may indicate that the MJO‐response in the momentum forcing of DE3(2) during winter months in our SD‐WACCMX simulations is underestimated.

Similar to Figure 9, the amplitudes of MJO‐forcing in Figures 10 and 11 show that between advection and GWD, advection is the dominant nonclassical momentum forcing term contributing to the MJO‐response in all three tidal components and the role of advection is generally larger in summer than in winter. Also, the overall double‐peak structure of the MJO‐response amplitudes as a function of MJO‐phases and its seasonal modulation with respect to MJO‐phases 1–4 and 5–7 resembles the modulation of the similar double‐peak structure in the MJO‐response amplitudes in the advection terms as well as the classical terms. The amplitude of MJO‐forcing in GWD largely follows the same winter to summer seasonal behavior in advection as a function of MJO‐phases. In addition, the double‐peak structure in the amplitude of the tidal MJO‐response as a function of MJO‐phases can be seen originated in the MJO‐response in radiative/latent forcing, especially in summer (Figure 6, Section 3.3), but the seasonal modulation of this peak structure becomes more evident in the MLT region (Figure 5, Section 3.2). The phase analysis of MJO‐response (MJO‐forcing) does not show an in‐phase relation between advection and GWD forcing in summer. This means that tidal advection and GWD MJO‐forcing can work against each other. However, advection is prominently larger than GWD. Briefly, the advection and GWD generally are in‐phase for DE3 in winter except the meridional wind advection and GWD for DE3(2) are out‐of‐phase in winter and in phase in summer. DW1 zonal wind advection and GWD are also in‐phase in summer while out‐of‐phase in winter. Meridional advection in DW1, however, is in‐phase with GWD in winter and out‐of‐phase in summer. Overall, the zonal momentum budget is two times as big as the meridional momentum budget for DE3 tidal components while they are comparable for DW1 tides, and advection and GWD together with Coriolis and pressure gradient force play significant roles in transporting MJO‐response to tides in the MLT region.

## 5 Conclusions

Our findings for the physical mechanisms that transmit the MJO into the MLT diurnal tides can be summarized as follows.

1. The statistical characteristics of the diurnal tidal MJO‐response as a function of MJO‐phases were extracted from Hovmoeller's analysis of SABER and SD‐WACCMX. Observed and modeled amplitude and phase modulations of the MJO‐response as a function of the eight MJO‐phases/locations agree reasonably well with each other.
2. The double‐peak structure in amplitude of the tidal MJO‐response shows seasonal variation from winter to summer as a function of MJO‐phases (1–4 & 5–7). In summer, the symmetric components (equatorial mode) of DW1(1) and DE3(1) show a comparable MJO‐response while the antisymmetric component (non‐equatorial mode) of DE3(2) shows a smaller MJO‐response than the equatorial modes. In winter, when the MJO is more active, the DE3(2) shows a comparable response to DE3(1) and DW1(1). The phase of the MJO‐response for DE3(2) also varies from winter to summer as a function of MJO‐phases.
3. The phase of the MJO‐response in radiative and latent heating is generally not consistent with that of the observed MLT tides, while the amplitude modulation of the tidal MJO‐response as a function of MJO‐phases shows similarities with either latent or radiative forcing or both for all tidal components (with double peak structures). The amplitude modulation of the MJO‐response in radiative and latent tidal heating is comparable in NH winter and NH summer even if MJO is most active during NH winter season. Additionally, the phases of the MJO‐response in latent and radiative tidal heating are generally consistent except for DE3(1). Hence, a connection exists between the MJO‐responses in radiative and latent tidal heating and their combined effects explain several characteristics of the magnitude of the MJO‐response in MLT tides as a function of MJO‐phases.
4. The MJO‐response diagnostics in tropo/stratospheric winds from MERRA2 shows a larger response in the zonal winds than in the meridional winds. The tropospheric MJO‐signal in zonal wind anomalies shows eastward propagating enhanced MJO‐anomalies with respect to longitudes. Interestingly, the MJO‐response in the zonal wind anomalies of the stratosphere shows a longitudinally independent global signal, which changes eastward to westward depending on MJO‐phases/locations. The modified SD‐WACCMX run with wind filtering effect removed in the troposphere and stratosphere, overall, shows little impact on the characteristics of the MJO‐response in the unmodified model run. Consequently, tropospheric tidal forcing is more important up to the stratosphere in shaping the modulation of the tidal MJO‐response as a function of MJO‐phases.
5. The underlying mechanisms responsible for transmitting the MJO‐response from the stratosphere to the MLT region are the nonclassical forcing mechanisms including advection and GWD as well as the classical mechanisms Coriolis and pressure gradient force. The seasonal modulation in the amplitudes of the tidal MJO‐response is the same as the seasonal oscillation of the MJO‐response in zonal and meridional MLT winds (momentum budget). The zonal wind momentum forcing is twice as big as the meridional wind momentum forcing. Advection forcing is the most dominant nonclassical forcing among advection and GWD and is larger in the summer season than in the winter, which also resembles the overall MJO‐response in the MLT tides. The advection and GWD forcing can work together or against each other on MLT tides depending on their phase relationship with respect to the MJO‐phases in a given season.

## Supporting information

Supporting Information S1

## Data Availability

SABER level 2a, version 2.07 data are available throughout the NASA SPDF at http://spdf.gsfc.nasa.gov. The RMM MJO index is obtained from http://www.bom.gov.au/climate/mjo/. MERRA2 data are available at https://disc.gsfc.nasa.gov/datasets?keywords=merra-2&page=1. SD‐WACCMX has been run on Clemson University's Palmetto supercomputer. The model source code can be found here ( https://www2.hao.ucar.edu/modeling/waccm-x ).

## References

[jgrd57149-bib-0001] Alexander, M. J., Grimsdell, A. W., Stephan, C. C., & Hoffmann, L. (2018). MJO‐related intraseasonal variation in the stratosphere: Gravity waves and zonal winds. Journal of Geophysical Research: Atmospheres, 123(2), 775–788. 10.1002/2017JD027620

[jgrd57149-bib-0002] Andrews, D. G., Holton, J. R., & Leovy, C. B. (1987). Middle atmospheric dynamics (p. 489). Academic Press.

[jgrd57149-bib-0003] Chang, L. C., Lin, C. H., Yue, J., Liu, J. Y., & Lin, J. T. (2013). Stationary planetary wave and nonmigrating tidal signatures in ionospheric wave 3 and wave 4 variations in 2007‐2011 FORMOSAT‐3/COSMIC observations. Journal of Geophysical Research: Space Physics, 118(10), 6651–6665. 10.1002/jgra.50583

[jgrd57149-bib-0004] Dhadly, M. S., Emmert, J. T., Drob, D. P., McCormack, J. P., & Niciejewski, R. J. (2018). Short‐term and interannual variations of migrating diurnal and semidiurnal tides in the mesosphere and lower thermosphere. Journal of Geophysical Research: Space Physics, 123(8), 7106–7123. 10.1029/2018JA025748

[jgrd57149-bib-0005] Eckermann, S. D., Rajopadhyaya, D. K., & Vincent, R. A. (1997). Intraseasonal wind variability in the equatorial mesosphere and lower thermosphere: Long‐term observations from the central Pacific. Journal of Atmospheric and Solar‐Terrestrial Physics, 59(6), 603–627. 10.1016/s1364-6826(96)00143-5

[jgrd57149-bib-0006] Fritts, D. C., & Alexander, M. J. (2003). Gravity wave dynamics and effects in the middle atmosphere. Reviews of Geophysics, 41(1), 1–64. 10.1029/2001RG000106

[jgrd57149-bib-0007] Gan, Q., Oberheide, J., Yue, J., & Wang, W. (2017). Short‐term variability in the ionosphere due to the nonlinear interaction between the 6 day wave and migrating tides. Journal of Geophysical Research: Space Physics, 122(8), 8831–8846. 10.1002/2017JA023947

[jgrd57149-bib-0008] Gasperini, F., Liu, H., & Mcinerney, J. (2020). Preliminary evidence of Madden‐Julian oscillation effects on ultra‐fast tropical waves in the thermosphere. Journal of Geophysical Research: Space Physics, 125, 1–47, e2019JA027649. 10.1029/2019JA027649

[jgrd57149-bib-0009] Gelaro, R., McCarty, W., Suárez, M. J., Todling, R., Molod, A., Takacs, L., et al. (2017). The modern‐era retrospective analysis for research and applications, version 2 (MERRA‐2). Journal of Climate, 30(14), 5419–5454. 10.1175/JCLI-D-16-0758.1 32020988 PMC6999672

[jgrd57149-bib-0010] Hagan, M. E., Chang, J. L., & Avery, S. K. (1997). Global‐scale wave model estimates of nonmigrating tidal effects. Journal of Geophysical Research, 102(14), 16439–16452. 10.1029/97JD01269

[jgrd57149-bib-0011] Hagan, M. E., & Forbes, J. M. (2002). Migrating and nonmigrating diurnal tides in the middle and upper atmosphere excited by tropospheric latent heat release. Journal of Geophysical Research, 107(24), 1–15. 10.1029/2001JD001236

[jgrd57149-bib-0012] Hagan, M. E., Maute, A., & Roble, R. G. (2009). Tropospheric tidal effects on the middle and upper atmosphere. Journal of Geophysical Research, 114(1), 1–6. 10.1029/2008JA013637

[jgrd57149-bib-0013] Immel, T. J., Mende, S. B., Hagan, M. E., Kintner, P. M., & England, S. L. (2009). Evidence of tropospheric effects on the ionosphere. Eos, Transactions, American Geophysical Union, 90(9), 69–70. 10.1029/2009EO090001

[jgrd57149-bib-0014] Immel, T. J., Sagawa, E., England, S. L., Henderson, S. B., Hagan, M. E., Mende, S. B., et al. (2006). Control of equatorial ionospheric morphology by atmospheric tides. Geophysical Research Letters, 33(15), 2–5. 10.1029/2006GL026161

[jgrd57149-bib-0015] Jones, M., Forbes, J. M., & Hagan, M. E. (2016). Solar cycle variability in mean thermospheric composition and temperature induced by atmospheric tides. Journal of Geophysical Research: Space Physics, 121(6), 5837–5855. 10.1002/2016JA022701

[jgrd57149-bib-0016] Jones, M., Forbes, J. M., Hagan, M. E., & Maute, A. (2014). Impacts of vertically propagating tides on the mean state of the ionosphere‐thermosphere system. Journal of Geophysical Research: Space Physics, 119(3), 2197–2213. 10.1002/2013JA019744

[jgrd57149-bib-0017] Jones, M., Forbes, J. M., & Sassi, F. (2019). The effects of vertically propagating tides on the mean dynamical structure of the lower thermosphere. Journal of Geophysical Research: Space Physics, 124(8), 7202–7219. 10.1029/2019JA026934

[jgrd57149-bib-0018] Kumari, K., & Oberheide, J. (2020). QBO, ENSO, and solar cycle effects in short‐term nonmigrating tidal variability on planetary wave timescales from SABER—An information‐theoretic approach. Journal of Geophysical Research: Atmospheres, 125(6), 1–23. 10.1029/2019JD031910

[jgrd57149-bib-0019] Kumari, K., Oberheide, J., & Lu, X. (2020). The tidal response in the mesosphere/lower thermosphere to the Madden‐Julian oscillation observed by SABER. Geophysical Research Letters, 47(16), e2020GL089172. 10.1029/2020GL089172

[jgrd57149-bib-0020] Li, J., & Lu, X. (2020). SABER observations of gravity wave responses to the Madden‐Julian oscillation from the stratosphere to the lower thermosphere in tropics and extratropics. Geophysical Research Letters, 47(23), e2020GL091014. 10.1029/2020gl091014

[jgrd57149-bib-0021] Lieberman, R. S. (1998). Intraseasonal variability of high‐resolution Doppler imager winds in the equatorial mesosphere and lower thermosphere. Journal of Geophysical Research, 103(D10), 11221–11228. 10.1029/98JD00532

[jgrd57149-bib-0022] Lieberman, R. S., Riggin, D. M., Ortland, D. A., Nesbitt, S. W., & Vincent, R. A. (2007). Variability of mesospheric diurnal tides and tropospheric diurnal heating during 1997–1998. Journal of Geophysical Research, 112(20), 1–17. 10.1029/2007JD008578

[jgrd57149-bib-0023] Lieberman, R. S., Riggin, D. M., Ortland, D. A., Oberheide, J., & Siskind, D. E. (2015). Global observations and modeling of nonmigrating diurnal tides generated by tide‐planetary wave interactions. Journal of Geophysical Research, 120(22), 11419–11437. 10.1002/2015JD023739

[jgrd57149-bib-0024] Liu, H. L. (2016). Variability and predictability of the space environment as related to lower atmosphere forcing. Space Weather, 14(9), 634–658. 10.1002/2016SW001450

[jgrd57149-bib-0025] Liu, H. L., Bardeen, C. G., Foster, B. T., Lauritzen, P., Liu, J., Lu, G., et al. (2018). Development and validation of the whole Atmosphere community climate model With thermosphere and ionosphere extension (WACCM‐X 2.0). Journal of Advances in Modeling Earth Systems, 10(2), 381–402. 10.1002/2017MS001232

[jgrd57149-bib-0026] Liu, H. L., Foster, B. T., Hagan, M. E., McInerney, J. M., Maute, A., Qian, L., et al. (2010). Thermosphere extension of the whole atmosphere community climate model. Journal of Geophysical Research, 115(12), 1–21. 10.1029/2010JA015586

[jgrd57149-bib-0027] Lu, X., Liu, A. Z., Oberheide, J., Wu, Q., Li, T., Li, Z. H., et al. (2011). Seasonal variability of the diurnal tide in the mesosphere and lower thermosphere over Maui, HI (20.7o N, 156.3o W). Journal of Geophysical Research, 116, D17103. 10.1029/2011JD015599

[jgrd57149-bib-0028] Lu, X., Liu, H. L., Liu, A. Z., Yue, J., McInerney, J. M., & Li, Z. (2012). Momentum budget of the migrating diurnal tide in the whole atmosphere community climate model at vernal equinox. Journal of Geophysical Research, 117(7), 1–14. 10.1029/2011JD017089

[jgrd57149-bib-0029] Madden, R. A., & Julian, P. R. (1972). Description of global‐scale circulation cells in the tropics with a 40–50 day period. Journal of the Atmospheric Sciences, 29, 1109–1123. 10.1175/1520-0469(1972)029<1109:DOGSCC>2.0.CO;2

[jgrd57149-bib-0030] Madden, R. A., & Julian, P. R. (1994). Observations of the 40–50‐day tropical oscillation—A review. Monthly Weather Review, 122, 814–837. 10.1175/1520-0493(1994)122<0814:OOTDTO>2.0.CO;2

[jgrd57149-bib-0031] Marsh, D. R., Mills, M. J., Kinnison, D. E., Lamarque, J. F., Calvo, N., & Polvani, L. M. (2013). Climate change from 1850 to 2005 simulated in CESM1(WACCM). Journal of Climate, 26(19), 7372–7391. 10.1175/JCLI-D-12-00558.1

[jgrd57149-bib-0032] Nischal, N., Oberheide, J., Mlynczak, M. G., Hunt, L. A., & Maute, A. (2017). Nonmigrating tidal impact on the CO_2_ 15 μm infrared cooling of the lower thermosphere during solar minimum conditions. Journal of Geophysical Research: Space Physics, 122(6), 6761–6775. 10.1002/2017JA024273

[jgrd57149-bib-0033] Nischal, N., Oberheide, J., Mlynczak, M. G., Marsh, D. R., & Gan, Q. (2019). Solar cycle variability of nonmigrating tides in the 5.3 and 15 μm infrared cooling of the thermosphere (100–150 km) from SABER. Journal of Geophysical Research: Space Physics, 124(3), 2338–2356. 10.1029/2018JA026356

[jgrd57149-bib-0034] Oberheide, J., & Forbes, J. M. (2008). Tidal propagation of deep tropical cloud signatures into the thermosphere from TIMED observations. Geophysical Research Letters, 35(4), 1–5. 10.1029/2007GL032397

[jgrd57149-bib-0035] Oberheide, J., Forbes, J. M., Häusler, K., Wu, Q., & Bruinsma, S. L. (2009). Tropospheric tides from 80 to 400 km: Propagation, interannual variability, and solar cycle effects. Journal of Geophysical Research Atmospheres, 114(23), 1–18. 10.1029/2009JD012388

[jgrd57149-bib-0036] Oberheide, J., Forbes, J. M., Zhang, X., & Bruinsma, S. L. (2011). Climatology of upward propagating diurnal and semidiurnal tides in the thermosphere. Journal of Geophysical Research: Space Physics, 116(11). 10.1029/2011JA016784

[jgrd57149-bib-0037] Oberheide, J., Hagan, M. E., Roble, R. G., & Offermann, D. (2002). Sources of nonmigrating tides in the tropical middle atmosphere. Journal of Geophysical Research, 107(21), 1–14. 10.1029/2002JD002220

[jgrd57149-bib-0038] Oberheide, J., Pedatella, N. M., Gan, Q., Kumari, K., Burns, A. G., & Eastes, R. W. (2020). Thermospheric composition O/N_2_ response to an altered meridional mean circulation during sudden stratospheric warmings observed by GOLD. Geophysical Research Letters, 47(1), 1–7. 10.1029/2019GL086313

[jgrd57149-bib-0039] Pedatella, N. M., Oberheide, J., Sutton, E. K., Liu, H., Anderson, J. L., & Raeder, K. (2016). Short‐term nonmigrating tide variability in the mesosphere, thermosphere, and ionosphere. Journal of Geophysical Research: Space Physics, 121, 3621–3633. 10.1002/2016JA022528

[jgrd57149-bib-0040] Powell, S. W. (2017). Successive MJO propagation in MERRA‐2 reanalysis. Geophysical Research Letters, 44(10), 5178–5186. 10.1002/2017GL07339

[jgrd57149-bib-0041] Rezac, L., Jian, Y., Yue, J., Russell, J. M., III, Kutepov, A., Garcia, R., et al. (2015). Validation of the global distribution of CO_2_ volume mixing ratio in the mesosphere and lower thermosphere from SABER. Journal of Geophysical Research: Oceans, 120, 12067–12081. 10.1002/2015JD023955

[jgrd57149-bib-0042] Russell, J. M., III, Mlynczak, M. G., Gordley, L. L., Tansock, J. J., Jr, & Esplin, R. W. (1999). Overview of the SABER experiment and preliminary calibration results (Vol. 3756). Optical Spectroscopic Techniques and Instrumentation for Atmospheric and Space Research III. 10.1117/12.366382

[jgrd57149-bib-0043] Sassi, F., McCormack, J. P., & McDonald, S. E. (2019). Whole atmosphere coupling on intraseasonal and interseasonal time scales: A potential source of increased predictive capability. Radio Science, 54, 913–933. 10.1029/2019RS006847

[jgrd57149-bib-0044] Tian, B., Ao, C. O., Waliser, D. E., Fetzer, E. J., Mannucci, A. J., & Teixeira, J. (2012). Intraseasonal temperature variability in the upper troposphere and lower stratosphere from the GPS radio occultation measurements. Journal of Geophysical Research, 117(15), 1–19. 10.1029/2012JD017715

[jgrd57149-bib-0045] Vergados, P., Liu, G., Mannucci, A. J., & Janches, D. (2018). Equatorial intraseasonal temperature oscillations in the lower thermosphere from SABER. Geophysical Research Letters, 45(20), 10893–10902. 10.1029/2018GL079467

[jgrd57149-bib-0046] Vitharana, A., Zhu, X., Du, J., Oberheide, J., & Ward, W. E. (2019). Statistical modeling of tidal weather in the mesosphere and lower thermosphere. Journal of Geophysical Research: Atmospheres, 124(16), 9011–9027. 10.1029/2019jd030573

[jgrd57149-bib-0047] Waliser, D. E., Moncrieff, M. W., Burridge, D., Fink, A. H., Gochis, D., Goswami, B. N., et al. (2012). The “year” of tropical convection (May 2008‐April 2010): Climate variability and weather highlights. Bulletin of the American Meteorological Society, 93(8), 1189–1218. 10.1175/2011BAMS3095.1

[jgrd57149-bib-0048] Wheeler, M., & Hendon, H. H. (2004). An all‐season real‐time multivariate MJO index: Development of an index for monitoring and prediction. Monthly Weather Review, 132(8), 1917–1932. 10.1175/1520-0493(2004)132<1917:AARMMI>2.0.CO;2

[jgrd57149-bib-0049] Wheeler, M., & Kiladis, G. N. (1999). Convectively coupled equatorial waves: Analysis of clouds and temperature in the wavenumber‐frequency domain. Journal of the Atmospheric Sciences, 56(3), 374–399. 10.1175/1520-0469(1999)056<0374:CCEWAO>2.0.CO;2

[jgrd57149-bib-0050] Wu, Q., Ortland, D. A., Killeen, T. L., Roble, R. G., Hagan, M. E., Liu, H. L., et al. (2008). Global distribution and interannual variations of mesospheric and lower thermospheric neutral wind diurnal tide: 1. Migrating tide. Journal of Geophysical Research: Space Physics, 113(5), 1–26. 10.1029/2007JA012542

[jgrd57149-bib-0051] Yang, C., Smith, A. K., Li, T., & Dou, X. (2018). The effect of the Madden‐Julian oscillation on the mesospheric migrating diurnal tide: A study using SD‐WACCM. Geophysical Research Letters, 45(10), 5105–5114. 10.1029/2018GL077956

[jgrd57149-bib-0052] Zhang, C. (2005). Madden‐Julian oscillation, Reviews of Geophysics, 43, RG2003. 10.1029/2004RG000158 PMC7375192 32879923

[jgrd57149-bib-0053] Zhang, X., Forbes, J. M., & Hagan, M. E. (2012). Seasonal‐latitudinal variation of the eastward‐propagating diurnal tide with zonal wavenumber 3 in the MLT: Influences of heating and background wind distribution. Journal of Atmospheric and Solar‐Terrestrial Physics, 78(79), 37–43. 10.1016/j.jastp.2011.03.005

