# Supplementary material for: Mechanism Studies of Madden‐Julian Oscillation Coupling Into the Mesosphere/Lower Thermosphere Tides Using SABER, MERRA‐2, and SD‐WACCMX
Source: J Geophys Res Atmos. 2021 Jul 2;126(13):e2021JD034595. doi: 10.1029/2021JD034595 (PMC8365709; doi:10.1029/2021JD034595)
Supplement: Supplementary file 1 — Supporting Information S1 [file JGRD-126-e2021JD034595-s001.docx]

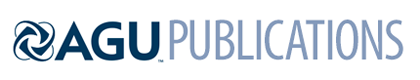


*[Journal of Geophysical Research: Atmospheres]*

Supporting Information for

**[Mechanism Studies of Madden-Julian-Oscillation Coupling into the Mesosphere/Lower Thermosphere Tides using SABER, MERRA-2, and SD-WACCMX]**

[Komal Kumari^1^, Haonan Wu^1^, Abigail Long^1^, Xian Lu^1^ and Jens Oberheide^1^]

[^1^Department of Physics and Astronomy, Clemson University, Clemson, SC 29631]

**Contents of this file**

Figures S1 to S13

**
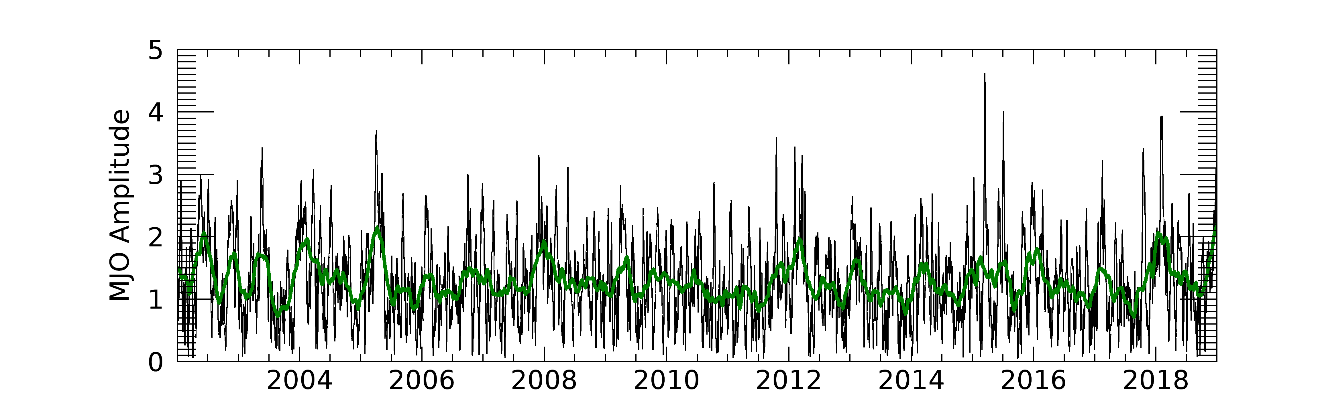
**

**Figure S1:** Daily RMM indices (also MJO amplitudes) are shown as black color and 91-day running mean of indices is shown as green color.


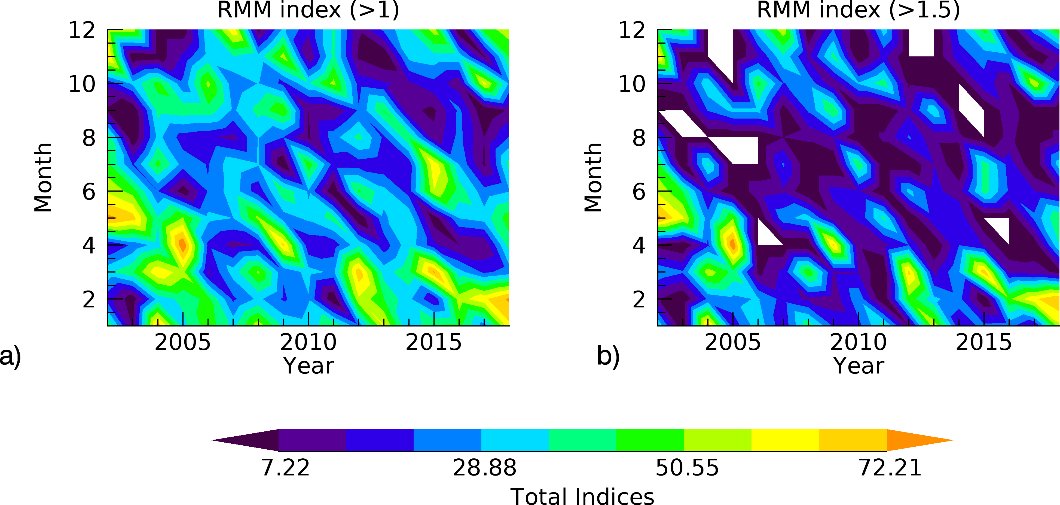


Figure S2: Monthly (y-axis) distribution of total (sum) RMM indices for the MJO active condition during 2002-2018 (x-axis), where a) RMM indices>1 and b) RMM indices>1.5 for 5 consecutive days is used for active-MJO condition.


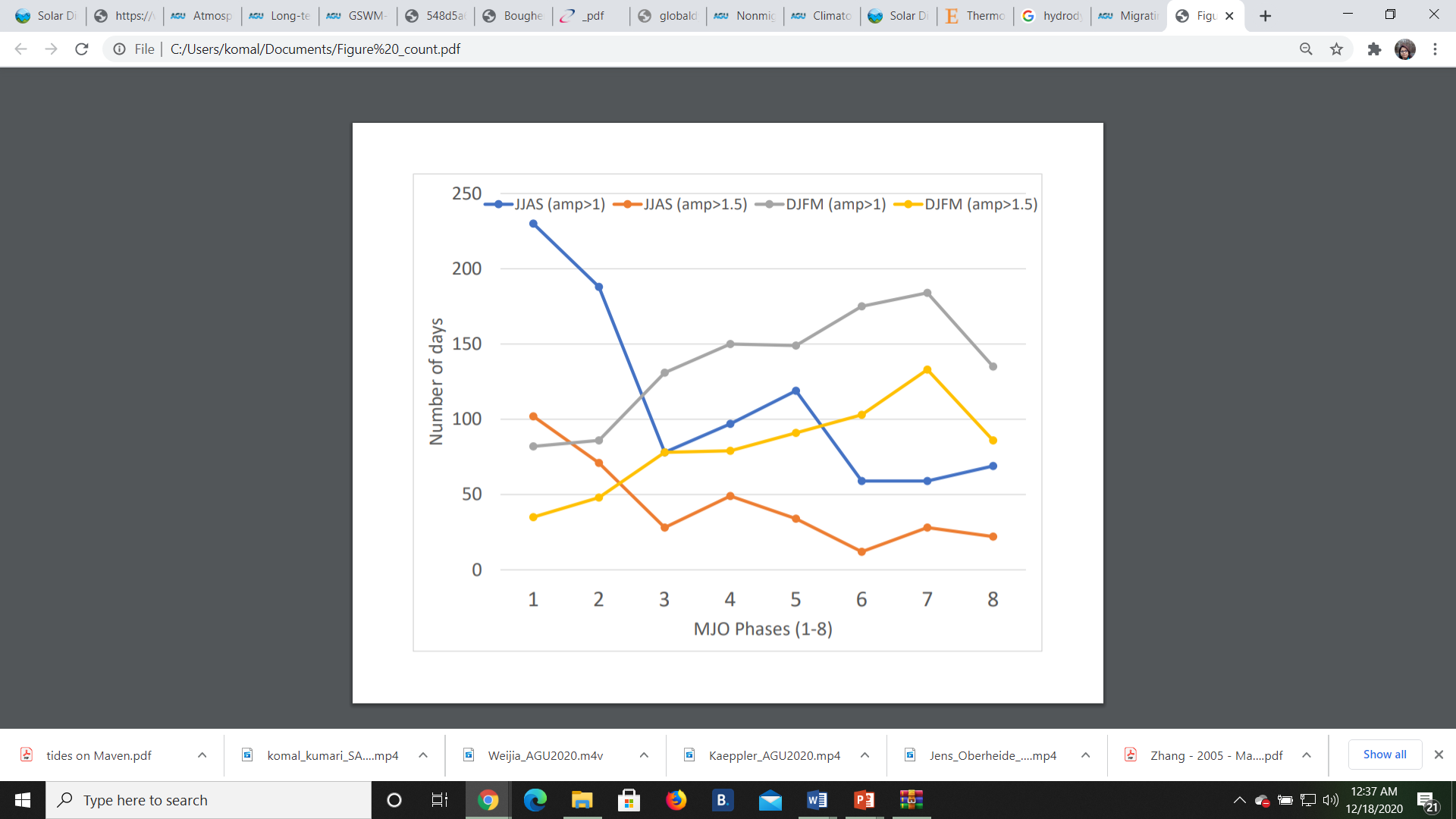


**Figure S3:** Number of data points (days) averaged for winter (DJFM) and summer (JJAS) seasons of 2004-2017 tidal anomalies in each MJO-phase/bin and for different active MJO conditions (amp>1 or amp>1.5).

**
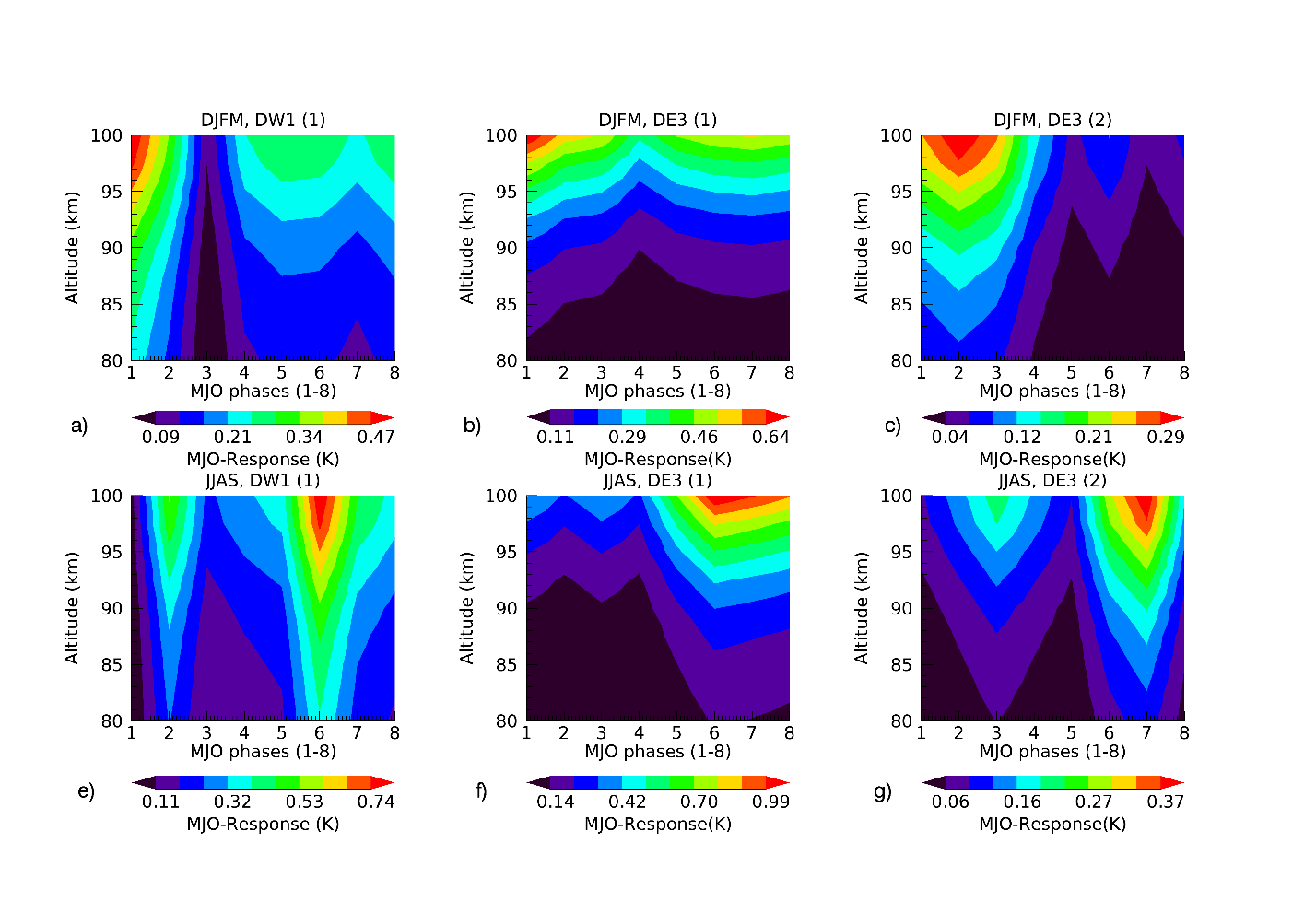
**

**Figure S4:** Amplitude of MJO-response in SABER MLT tidal HME components as a function of altitude and MJO-phases

**
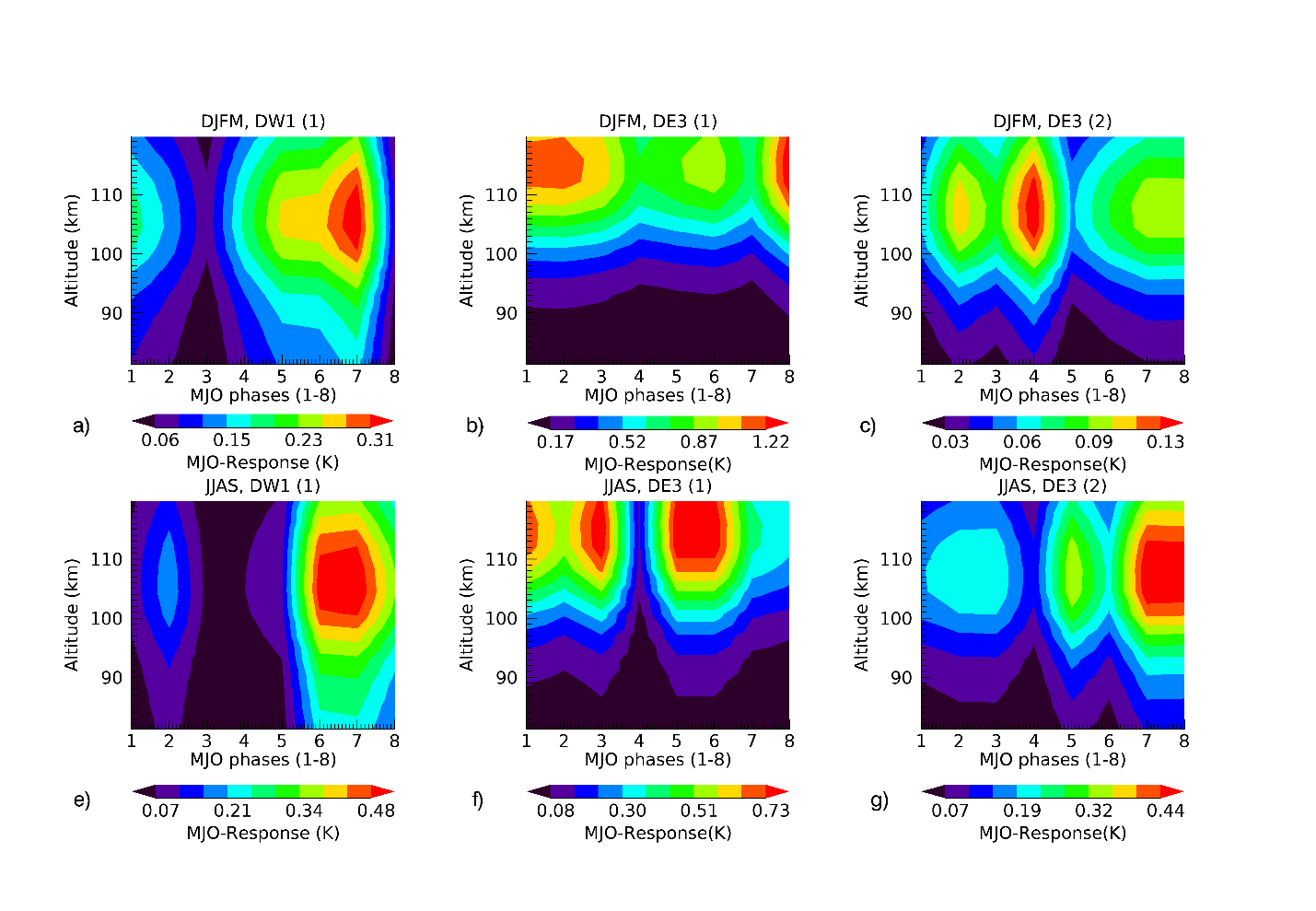
**

**Figure S5:** Same as S4, but for SD-WACCMX MLT tidal HME components.


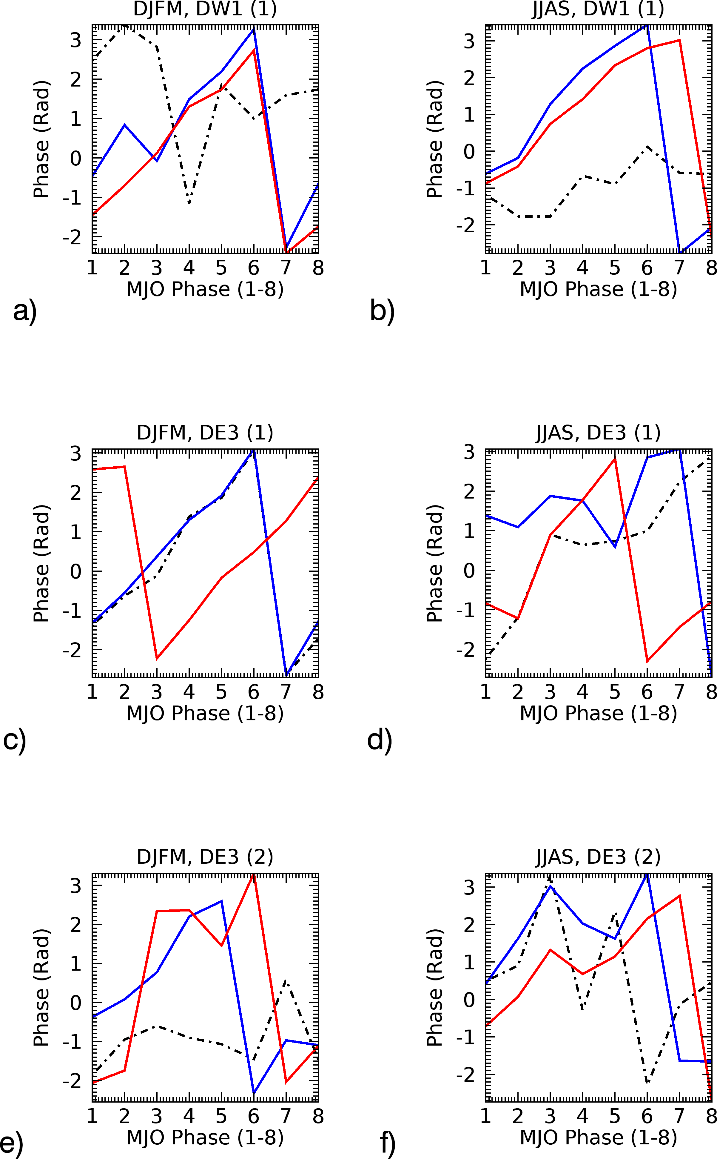


**Figure S6:** Phases of SABER MLT tidal MJO response (black lines, RMM>1.5) with the phases of the response in the radiative (red) and latent (blue) tidal heating (RMM>1).


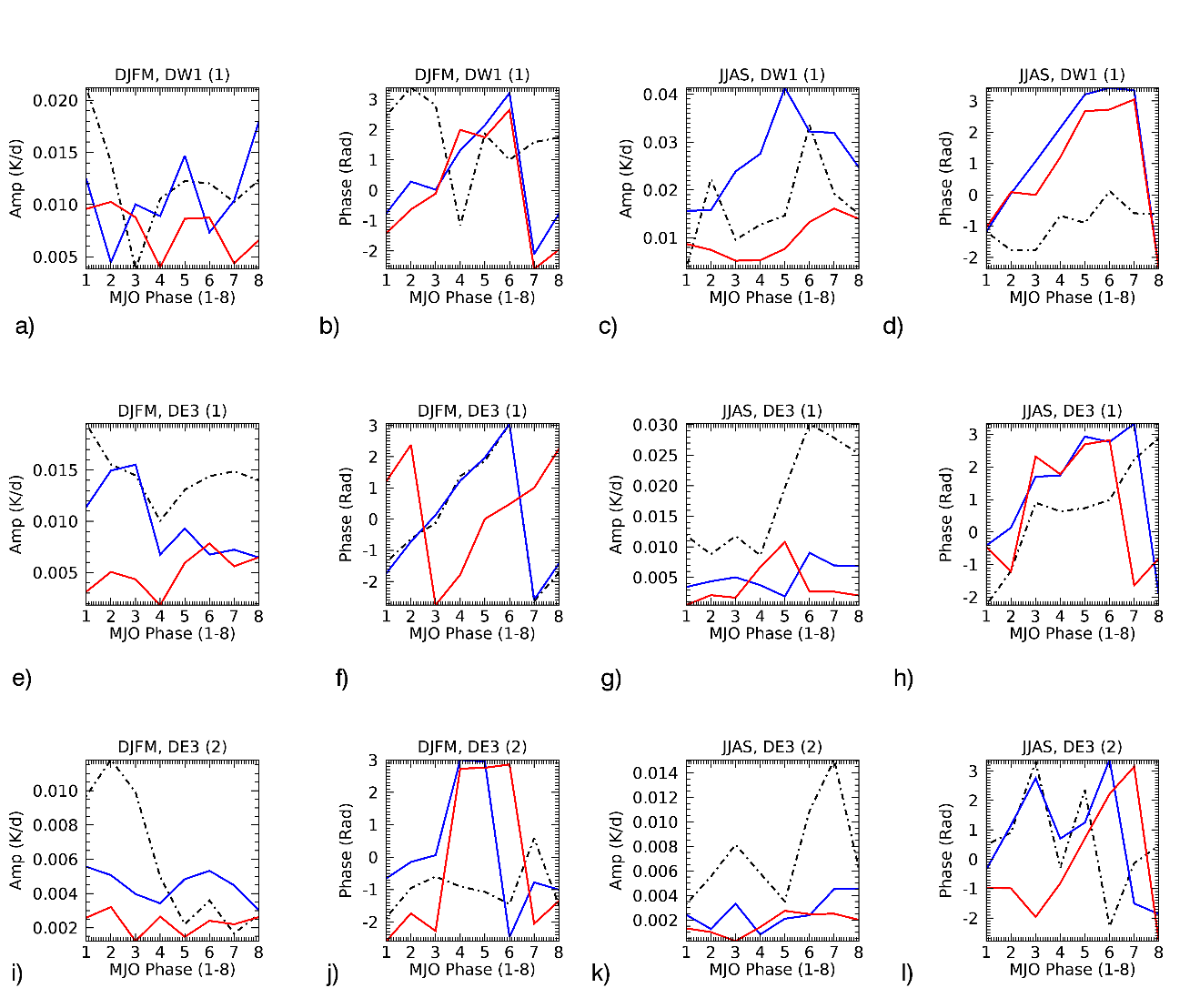


**Figure S7:** Same as Figure 6 (amplitude) and S6 (phases) but for both SABER tides and heating with RMM>1.5 condition for active-MJO days.


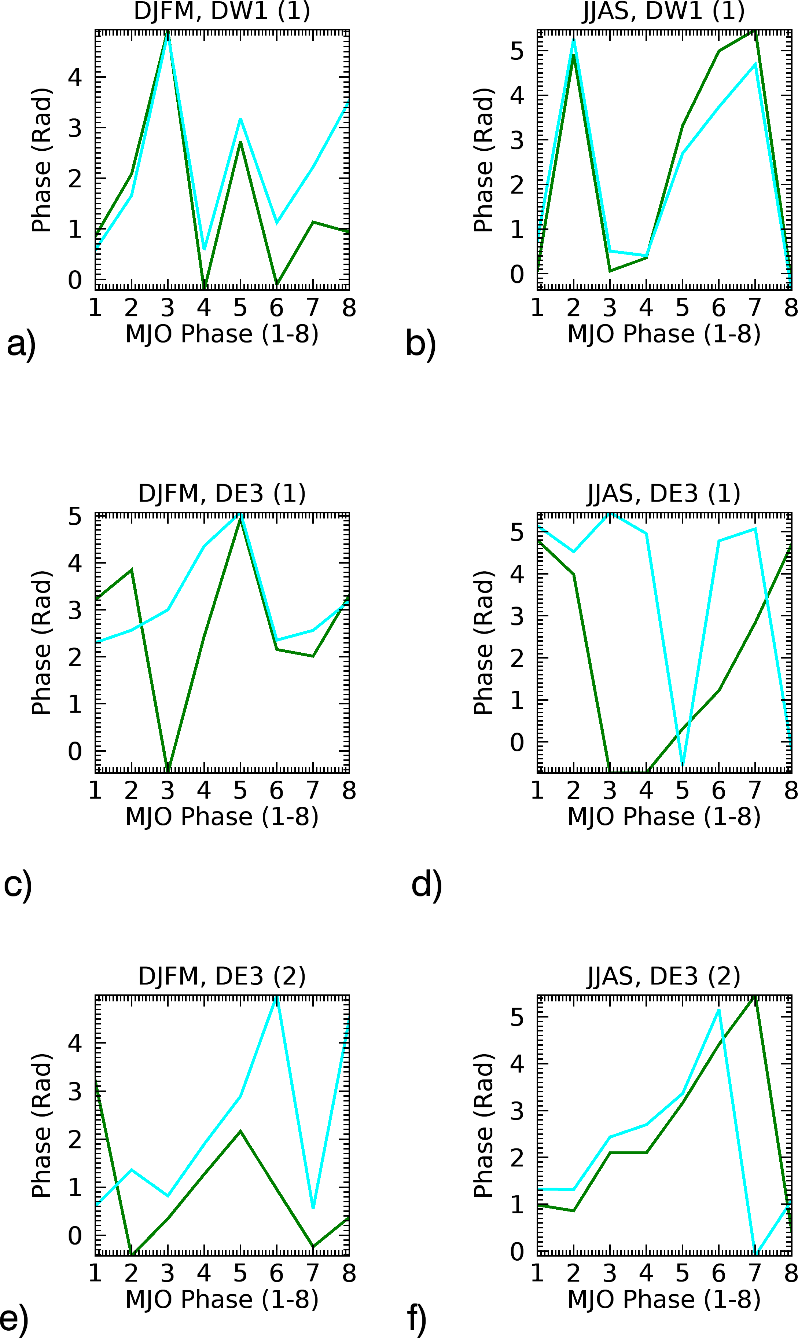


**Figure S8:** Same as S6, but for the phases of SD-WACCMX MLT tidal MJO-response with and without tropo/stratospheric wind filtering effect.


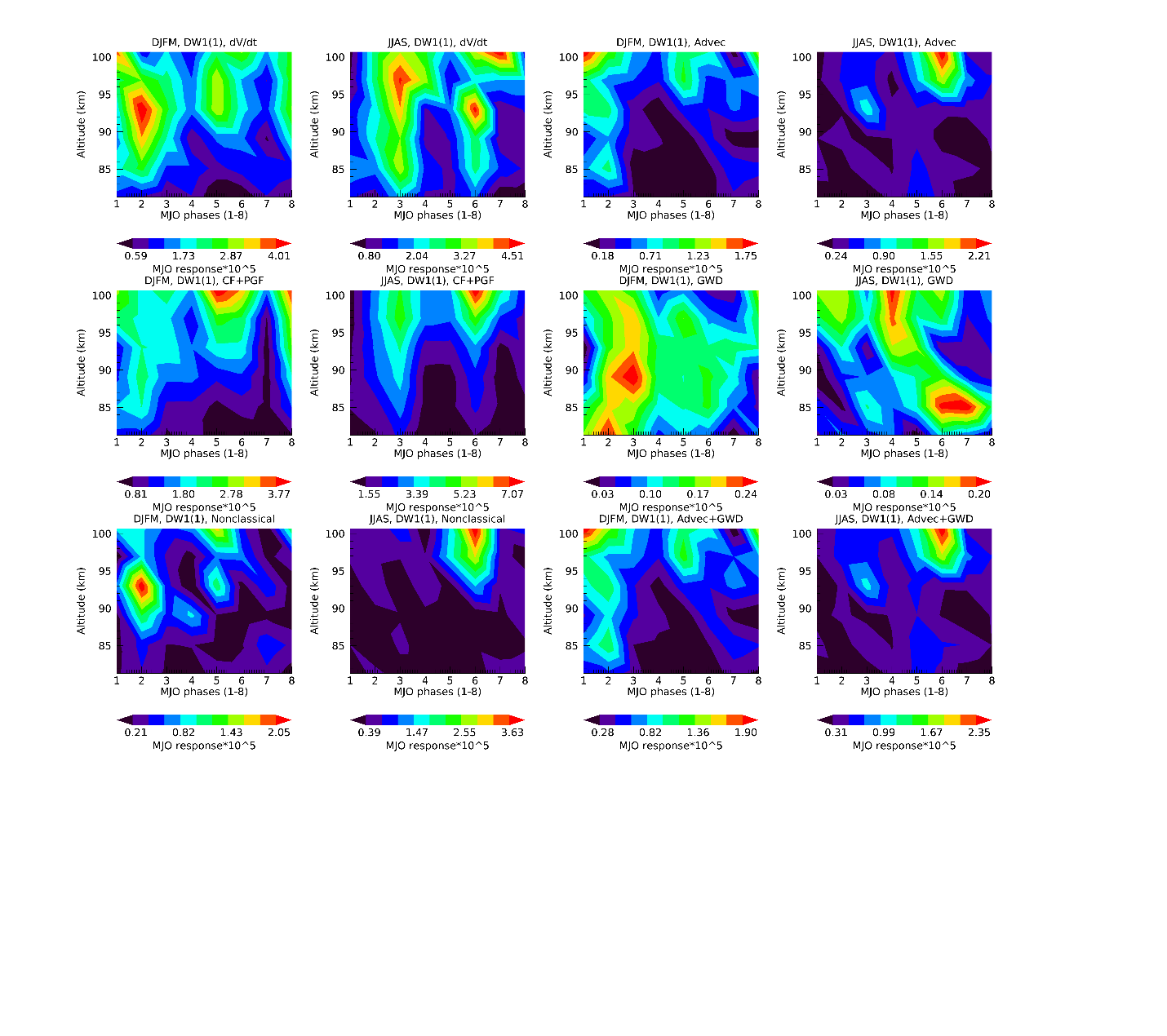


**Figure S9:** Same as Figure 9, but for DW1(1) meridional wind momentum budget

**
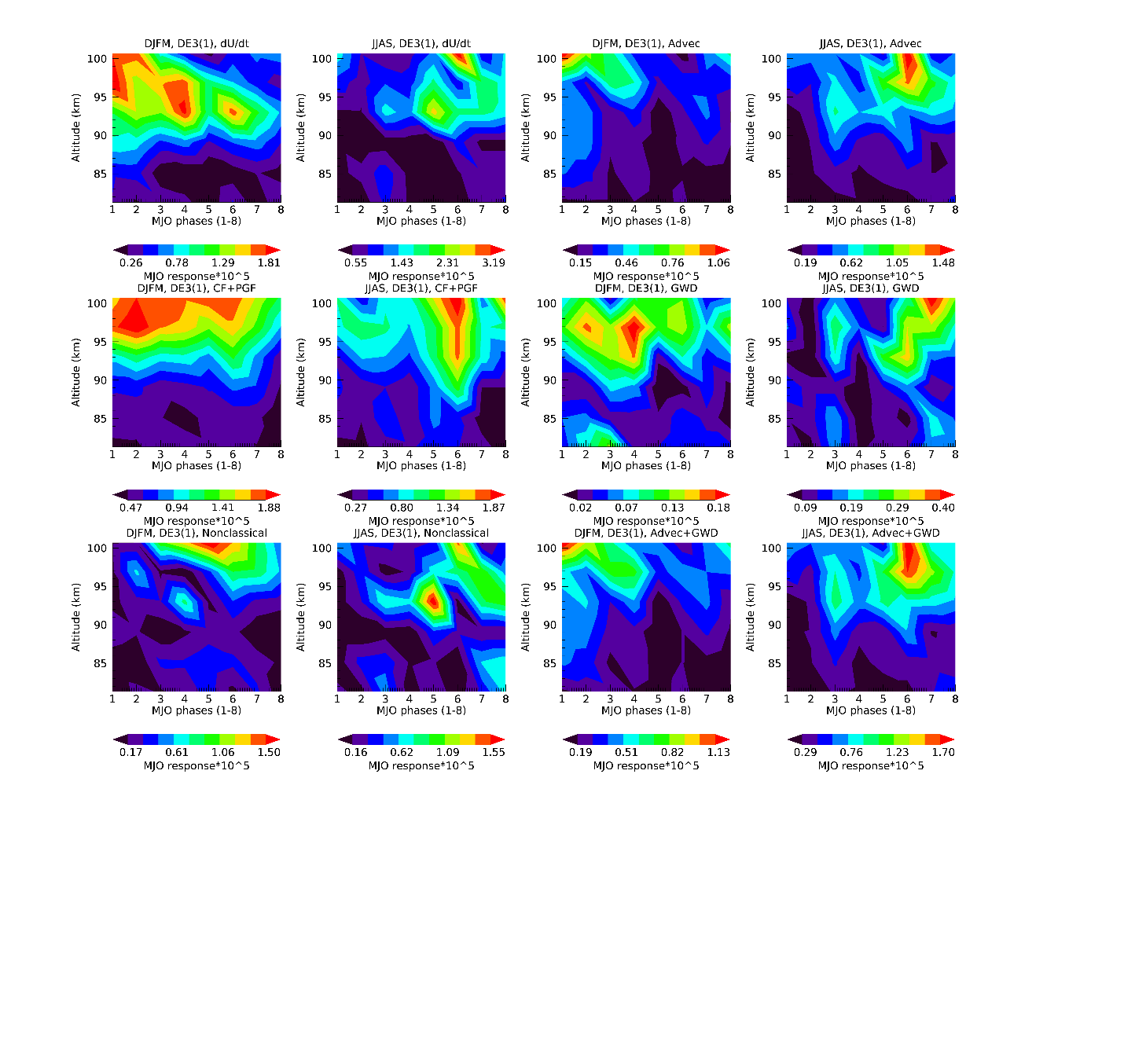
**

**Figure S10:** Same as Figure 9, but for DE3(1) zonal wind momentum budget

**
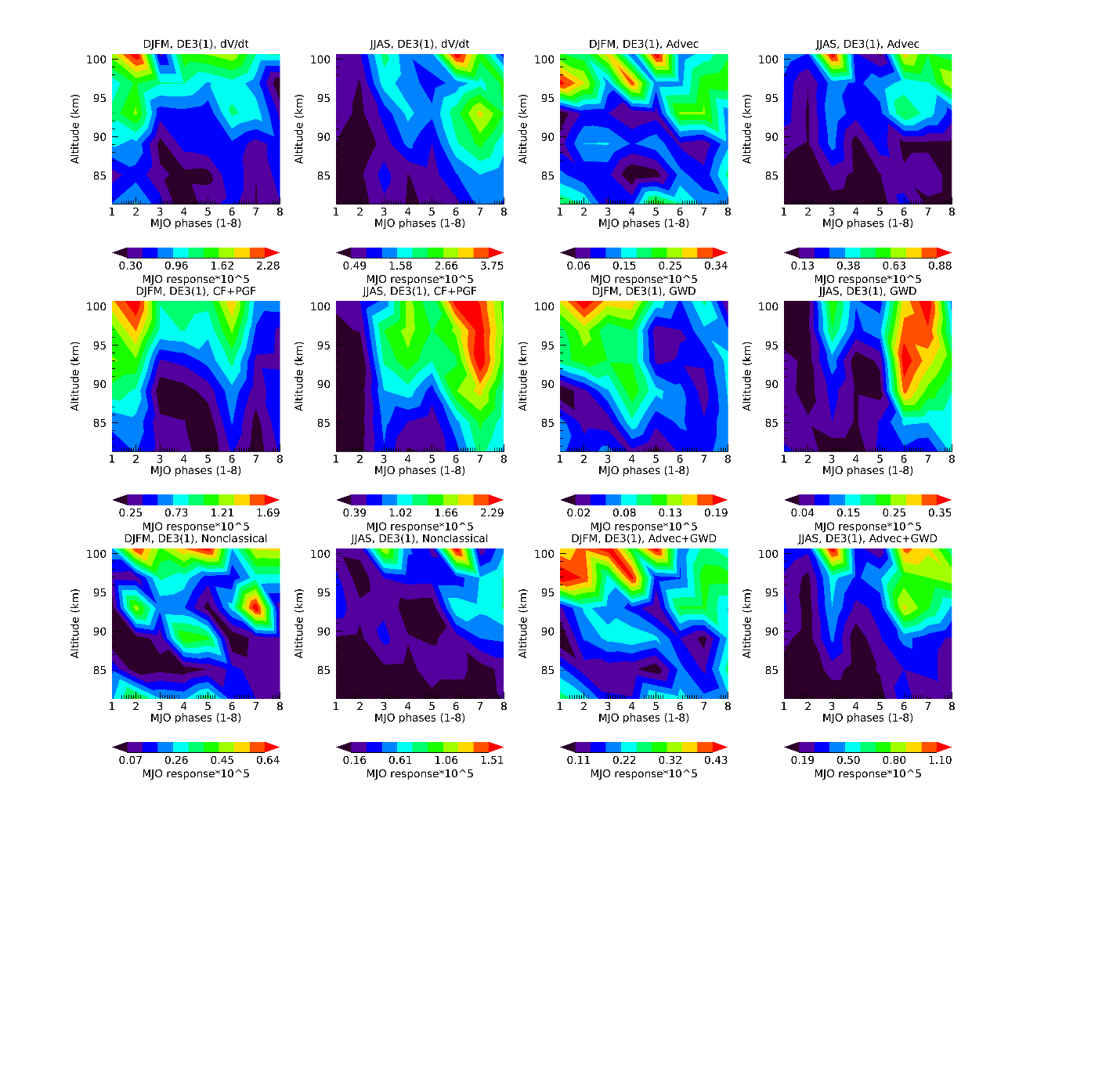
**

**Figure S11:** Same as Figure 9, but for DE3(1) meridional wind momentum budget

**
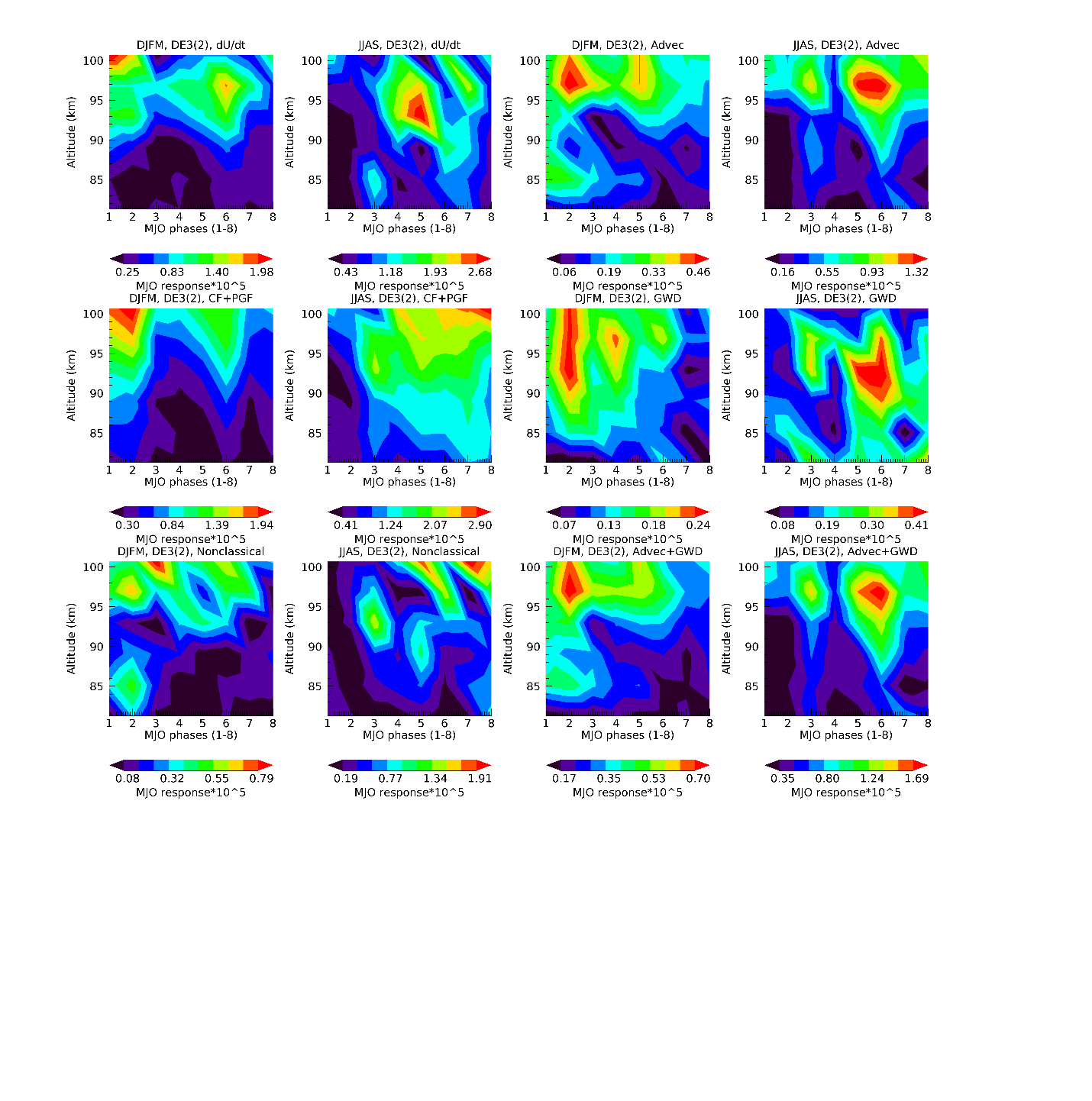
**

**Figure S12:** Same as Figure 9, but for DE3(2) zonal wind momentum budget

**
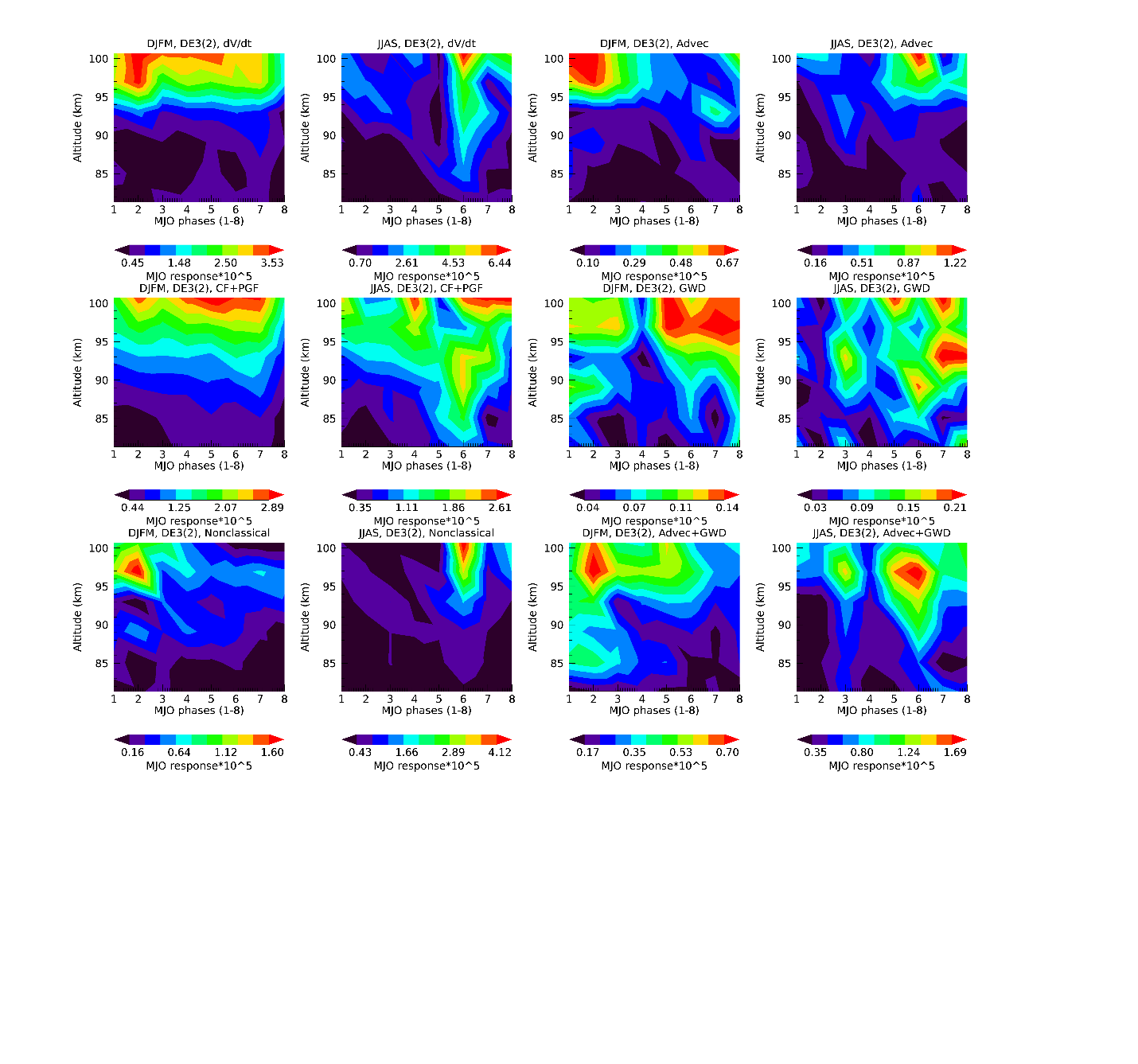
**

**Figure S13:** Same as Figure 9, but for DE3(2) meridional wind momentum budget
